# Bistable Insect‐Scale Jumpers with Tunable Energy Barriers for Multimodal Locomotion

**DOI:** 10.1002/advs.202404404

**Published:** 2024-07-07

**Authors:** Qingkai Guo, Yu Sun, Tianxiang Zhang, Shiyu Xie, Xuefeng Chen, Zhuang Zhang, Hanqing Jiang, Laihao Yang

**Affiliations:** ^1^ School of Mechanical Engineering Xi'an Jiaotong University Xi'an 710049 China; ^2^ School of Engineering Westlake University Hangzhou Zhejiang 310030 China; ^3^ Westlake Institute for Advanced Study Hangzhou Zhejiang 310024 China; ^4^ Research Center for Industries of the Future Westlake University Hangzhou Zhejiang 310030 China

**Keywords:** bistable structure, insect‐scale jumper, multimodal locomotion, tunable energy‐barrier

## Abstract

Drawing inspiration from the jumping mechanisms of insects (e.g., click beetles), bistable structures can convert slow deformations of soft actuating material into fast jumping motions (i.e., power amplification). However, bistable jumpers often encounter large energy barriers for energy release/re‐storage, posing a challenge in achieving multimodal (i.e., height/distance) and continuous jumps at the insect scale (body length under 20 mm). Here, a new offset‐buckling bistable design is introduced that features antisymmetric equilibrium states and tunable energy barriers. Leveraging this design, a Boundary Actuation Tunable Energy‐barrier (BATE) jumper (body length down to 15 mm) is developed, and transform BATE jumper from height‐jump mode (up to 12.7 body lengths) to distance‐jump mode (up to 20 body lengths). BATE jumpers can perform agile continuous jumping (within 300 ms for energy release/re‐storage times) and real‐time status detection is further demonstrated. This insect‐level performance of the proposed BATE jumper showcases its potential toward future applications in exploration, search, and rescue.

## Graphical abstract

In this innovative exploration, researchers unveil a novel insect‐scale jumper robot, featuring an offset‐buckling bistable design for tunable energy barriers. The study delves into the mechanics behind multimodal locomotion, showcasing the robot's capability to transition between height and distance jump modes, and introduces its ability to perform agile continuous jumps.

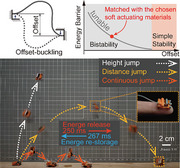

## 1 Introduction

Jumping, a locomotion strategy seen in animals, allows for overcoming obstacles, escaping predators, and exploring cluttered environments efficiently.^[^ ^1^, ^2^, ^3^ ^]^ Drawing inspiration from this natural phenomenon, the development of insect‐scale jumping robots seeks to mimic such agility and efficiency, holding promise for applications in exploration, inspection, search and rescue,^[^ ^4^, ^5^ ^]^ and even low‐gravity extraterrestrial environments.^[^ ^6^, ^7^, ^8^ ^]^ However, robots that utilize conventional rigid components for energy‐storage,^[^ ^9^, ^10^, ^11^, ^12^, ^13^ ^]^ such as motors and springs, although capable of achieving jumps of impressive heights, encounter significant hurdles when it comes to miniaturization. The state‐of‐the‐art in designing insect‐scale robots involves soft actuating materials.^[^ ^14^, ^15^, ^16^, ^17^, ^18^, ^19^ ^]^ Yet, these materials come with their own set of challenges, including low energy density (e.g., PVDF, DE actuators operated near resonance with small force and stroke output), slow response time (e.g., hyperelastic soft materials), and lengthy recovering periods (e.g., soft thermal actuators), all of which hinder their effectiveness in robotic jumping applications.^[^ ^20^, ^21^, ^22^ ^]^

**Figure 1:**
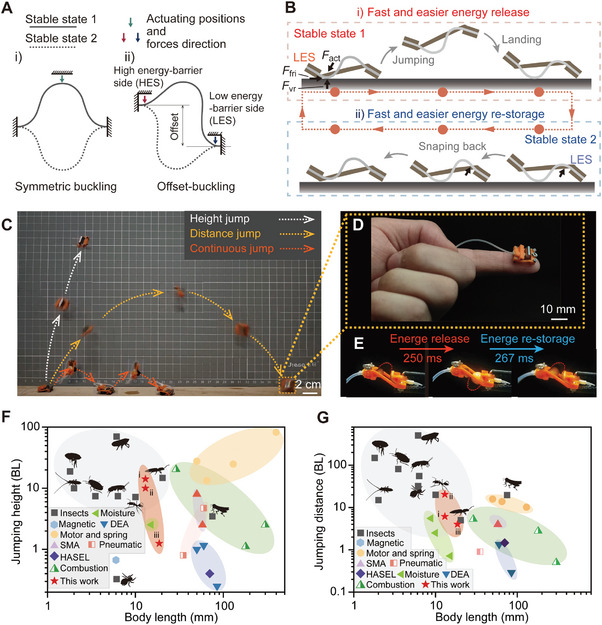
Concept of bistable offset‐buckling structure for insect‐scale robotic jumping. A) Bistable buckling beams with symmetric and antisymmetric equilibrium states, where the solid and dashed lines represent the two equilibrium states, and the colored arrows indicate the location and direction of the corresponding triggering forces. Under offset‐buckling, triggering from lower energy barrier (LES) facilitates easier snap‐through activation than the other side (HES). B) Energy release and re‐storage mechanism triggering form LES, with actuating force *F*_act_, frictional force *F*_fri,_ and vertical reaction force *F*_vr_. C) Video frames of the BATE jumper in height, distance, and continuous jumping modes. D) Photograph of the jumper on a human fingertip. E) Energy release and re‐storage time of the bistable structure in BATE jumper. F,G) Comparison of this work with various insects and previous jumping robots in terms of jumping heights/distances with respect to body length under height i), distance ii), and continuous iii) jumping modes.

Some insects such as click beetles^[^ ^23^ ^]^ and trap‐jaw ants,^[^ ^24^, ^25^ ^]^ despite their diminutive size (body length under 20 mm), can achieve legless jumps with remarkable speed and power by leveraging snapping mechanisms within their anatomy. Click beetles use a bistable spring‐and‐latches mechanism between their thorax and abdomen to store elastic energy. Upon triggering, the latch releases, and the stored elastic energy is rapidly released to generate a jump. Similarly, trap‐jaw ants leverage their powerful mandibles in a bistable manner to store and release energy, enabling them to propel themselves to heights orders of magnitude greater than their body length. Yet, there seems to be a gap in the development of inset‐scale robotic designs that are either bistable or multistable and capable of replicating these high‐power movements. Specifically, there's a scarcity of designs that effectively utilize soft actuating materials to produce the necessary force and stroke to overcome energy‐barrier, characterized by critical force *F*_crit_ and critical displacement *D*_crit_.^[^ ^26^, ^27^, ^28^ ^]^ Furthermore, the prevalent design in existing bistable jumping robots typically features a conventional symmetric bistable configuration^[^ ^29^, ^30^, ^31^, ^32^, ^33^, ^34^ ^]^(**Figure** **1**A‐i), which is effective for generating movement, usually results in a simple motion type and often requires extra external stimuli to control the jumping direction.^[^ ^35^, ^36^, ^37^ ^]^

Here, we present an advanced insect‐scale jumper through an offset‐buckling bistable design that enables tunable energy barriers, utilizing two antisymmetric equilibrium states for enhanced performance (Figure 1A‐ii). The offset between the low and high energy‐barrier sides (LES and HES, respectively) facilitates this tunability, improving the time efficiency of energy release and restorage, and permits exclusively forward friction during beam‐to‐ground interaction (Figure 1B). This unique configuration allows for dramatic reductions in the critical force *F*_crit_ and displacement *D*_crit_ required for actuation, achieving reductions of up to six and nine times, respectively, compared to conventional symmetric designs (see Supplementary Text 1, Figure S1, Movie S1, and S2, Supporting Information). Our Boundary Actuation Tunable Energy‐barrier (BATE) jumper, with a body length (BL) of just 15 mm, showcases multimodal locomotion, transitioning between height and distance modes, achieving leaps up to 12.7 and 20 BL, respectively (Figure 1C,D; Movie S3, Supporting Information). The BATE jumper can also execute continuous jumps spanning 4.2 BL, all within a swift 300 ms for energy release/re‐storage (Figure 1C,E). This level of performance, coupled with its miniature scale, positions the BATE jumper beyond the capabilities of many existing insects and robots (Figure 1F,G; Figure S2, Tables S1, and S2, Supporting Information), highlighting its potential for innovative applications.

## 2 Results and Discussion

### 2.1 Design Rationale for Instability Response and Tunable Energy Barriers

**Figure 2:**
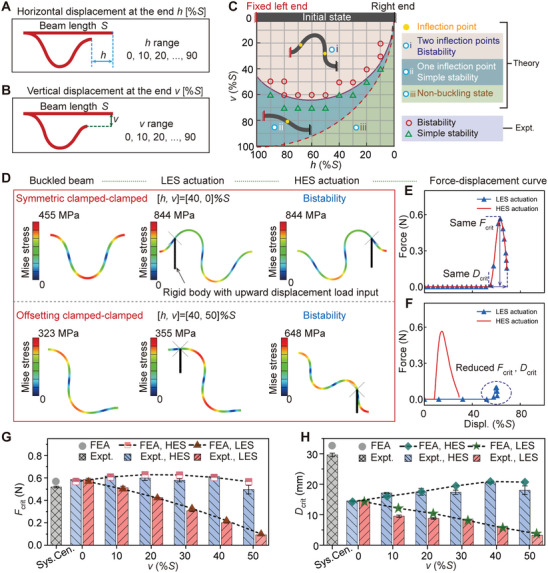
Rationale of instability response and tunable energy‐barrier of offset‐buckling designs. A,B) Governing geometrical parameters of the buckling beam designs. A) Horizontal displacement at the end, *h*, and B) vertical displacement at the end, *v*. C) Instability phase diagram of the buckling beam using the elastica solution and experiments. D,E, and F) Configuration and stress distribution of the buckled beam in different states, and corresponding force‐displacement curves. G,H) Analysis of *F*_crit_ and *D*_crit_ for varying vertical displacement *v*.

In our study, we combined theoretical calculations, finite element simulations, and experimental characterization to delve into the instability response and energy‐barrier variations across different offsetting configurations. The buckling behavior is mainly dictated by two variables: i) the horizontal displacement at the beam's end, *h*, expressed as a percentage of the beam length, *S*, ranging from 0% to 90% (*S* is fixed at 100 mm for this analysis) (**Figure** **2**A), and ii) the vertical displacement or offset distance at the beam's end, *v*, also measured as a percentage of *S* (Figure 2B).

We began our investigation by applying the elastica solution^[^ ^38^, ^39^, ^40^, ^41^ ^]^ to analyze the static deformation of buckling beams (see Supplementary Text 2, Supporting Information), aiming to pinpoint the snap‐through instability at various offset positions. Our model utilized a spring steel beam 50 µm in thickness and 12.7 mm in width, with Young's modulus *E* of 194 GPa and a Poisson's ratio of 0.3, consistent with our experimental parameters. The analysis commenced with determining the form of a single buckling beam unit (Figure S3A, Supporting information) and extended to examining the shape of offset‐buckling beams, modeled as a segment of the combined elastica curves from three beam units (Figure S3B,C, Supporting information). Neglecting axial and shear deformations, the length coordinate (*s*) and the Cartesian coordinates (*x*, *y*) of the beam unit's elastica can be expressed by the following equations,

(1) $$\begin{array}{ccc} s\; & = & \sqrt{\frac{EI}{N}}\;\left[K\left(\sin\frac{\alpha }{2},\frac{\pi }{2}\right)-K\left(\sin\frac{\alpha }{2},\varphi \right)\right],\varphi \;\\ & & =\sin^{-1}\left(\frac{\sin\frac{1}{2}\theta }{\sin\frac{1}{2}\alpha }\right)\; \end{array}$$

(2) $$\begin{array}{ccc} x & = & \sqrt{\frac{EI}{N}}\left[2F\left(\sin\frac{1}{2}\alpha ,\frac{1}{2}\pi \right)-2F\left(\sin\frac{1}{2}\alpha ,\varphi \right)\right.\\ & & \left.-K\left(\sin\frac{1}{2}\alpha ,\frac{1}{2}\pi \right)+K\left(\sin\frac{1}{2}\alpha ,\varphi \right)]\right. \end{array}$$

(3) $$y\;=\sqrt{\frac{2EI}{N}\left(\cos\theta -\cos\alpha \right)}\;,-\alpha \leq \theta \leq \alpha$$

in which *K* and *F* are the elliptic integrals of the first and second kind, respectively, α and θ are the shape parameters of the beam unit in post‐buckling state (Figure S3A‐iii, Supporting information).

We analyzed configurations across a range of angles α from 10° to 100°, detailing our computational process in Figure S4 (Supporting Information). By connecting critical points for each α, we outlined the instability phase diagram in Figure 2C, in which three distinct regions are revealed: region i (beige), where the buckling beams feature two inflection points indicating two stable states; region ii (cyan), where the beams exhibit a single stable state with one inflection point; and region iii (green), characterized by beams in tension, which falls outside the scope of this study. Following the theoretical analysis, we conducted experimental validations under identical boundary conditions, using a grid platform to precisely place the beam's right end at points along the instability demarcation line. The experimental outcomes closely matched our model's predictions, underscoring the model's accuracy in forecasting buckling beam behaviors.

To better characterize the triggering process as well as the *F*_crit_ and *D*_crit_ of the bistable beams, we conducted finite element analyses using the commercial package ABAQUS/Standard. We specifically chose a horizontal displacement (*h*) at 40% of the beam length (*S*), targeting this parameter to investigate the influence of vertical offsets, as beams at this *h* value offer ample opportunities for offset adjustments. In our boundary‐actuation setup, we applied unidirectional force and displacement inputs to the bistable beam at a horizontal distance of 10% *S* from the left and right ends of beam (see the black rigid in Figure 2D). Figure 2D shows, from left to right, the configuration and the stress distribution of the buckled beam went through three states: i) buckling without external force, ii) actuated to the critical state from the LES, and iii) similarly from the HES. For a symmetrically buckled beam (with [*h*, *v*] = [40, 0]*%S*), we noted identical *F*_crit_ (≈0.6 N) and *D*_crit_ (≈14.5 mm) from both left and right sides (Figure 2E). In contrast, the offset‐buckling beam (with [*h*, *v*] = [40, 50]*%S*) exhibited a substantial decrease in *F*_crit_ (≈0.1 N, an 83% reduction) and *D*_crit_ (≈3.3 mm, a 77% reduction) when triggered from the LES, while triggering from HES remained largely unaffected (Figure 2F), highlighting the significant impact of vertical offsets on the ease of actuation.

We further analyzed how varying vertical offsets (*v* from 0% to 50% of the beam length *S*) affect the critical force *F*_crit_ (Figure 2G) and displacement *D*_crit_ (Figure 2H). For symmetrical buckling, central actuators yielded large forces and displacements with *F*_crit_ ≈0.52 N and *D*_crit_ ≈30 mm. The *F*_crit_ and *D*_crit_ for triggering from the high‐energy barrier side (HES) remained almost insensitive as increased offset, while from the low‐energy barrier side (LES), both decreased monotonically with slopes of ≈0.9 for the *F*_crit_ and ≈0.3 for *D*_crit_, significantly easing actuation. Finite element simulations confirmed these trends, showing our offset‐buckling design reduces *F*_crit_ and *D*_crit_ considerably, enhancing the efficiency of bistable mechanism actuation.

### 2.2 Design and Height‐Jump Performance of the BATE Jumper

**Figure 3:**
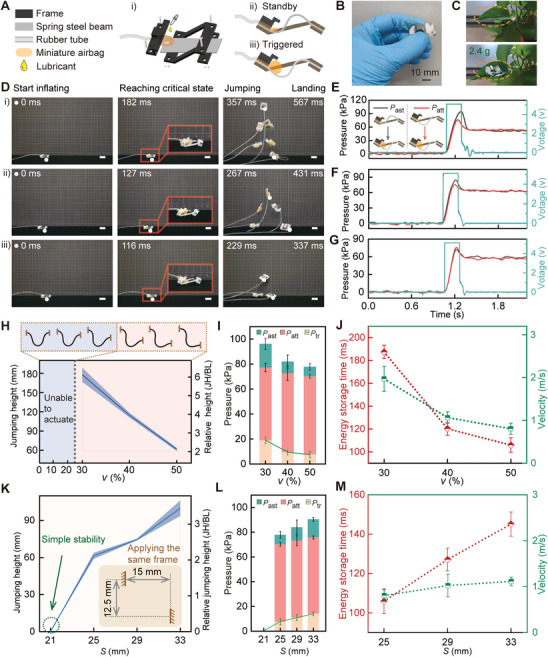
Structural design and height‐jump performances of the BATE jumper. A) The BATE jumper consists of four main parts: frame, spring steel beam, air tube, and miniature airbag. When the air pressure in the system exceeds a critical value, the robot is triggered from standby to activated. B,C) Physical illustration of the BATE jumper shows its length of 28 mm and a frame of 2.4 g. D,E,F, and G) Video frames of the robot jumping in 3 configurations (*v* ranging from 30% to 50% of *S*) with corresponding changes in internal air pressure and air pump supply voltage. H,I, and J) Jumping height performance, air pressure, and energy storage time with initial jump speed for different offset distances, *v*. K,L, and M) Effect of the buckling beam length *S* on the robot's jumping performance for the same external frame. Scale bar, 20 mm.

To showcase our tunable energy‐barrier design's utility in soft actuator‐based insect‐scale jumping robots, we developed the BATE jumper (**Figure** **3**A), incorporating a 3D‐printed polylactic acid rigid frame and a spring steel beam for the bistable mechanism. We integrated a mini airbag actuator, connected via a silicone tube embedded in the frames’ edge, and applied the dimethyl silicone oil lubricant to reduce friction between the airbag and beam, enhancing jumping performance (Figure S5, Supporting Information shows the effect of the lubricant on the jumping height of the robot). Figure 3A(ii,iii) shows the standby and activated states of the BATE jumper, demonstrating its compact and lightweight design (28 mm × 21 mm × 11 mm, weight 2.4 g), which ensures it can rest on Epipremnum aureum leaves without causing damage (Figure 3B,C).

The BATE jumper's jumping dynamics are captured in video frames (Figure 3D), showing jumping in varying configurations (vertical offsets *v* from 30% to 50% of the beam length *S*). The jumping process consists of four phases: inflation start, reaching the critical point, jumping, and landing. An air pump (5 V) activated for 210 s (cyan curves in Figure 3E–G) induces airbag inflation, leading to pressurization that triggers the snap‐through and releasing energy. As the vertical offset *v* increases, the jumping height decreases. The BATE jumper's design, with its center of gravity toward the tail, causes a significant head deflection (≈90°) during jumping, resulting in an upward‐facing posture upon landing. Figure 3E–G depicts the internal air pressures (*P*_ast_ and *P*_att_) during inflation for these three scenarios. *P*_ast_ and *P*_att_ represent air pressure changes from the steady state and the triggered state (inset of Figure 3E), respectively, with the additional air pressure (*P*_tr_) required for activation. As the jumping height decreases, the corresponding *P*_tr_ decreases (see smaller gap between *P*_ast_ and *P*_att_), indicating a smaller energy input is needed for lower jumps.

Figure 3H demonstrates the BATE jumper's vertical jumping heights across different configurations, with *h* fixed at 40% of beam length *S*. For vertical offset *v* from 0% to 20% of *S*, the airbag couldn't trigger the buckling beam due to a high energy barrier (Figure S6, Supporting information), resulting in ineffective force application as the airbag expanded in unintended directions. However, for offsets *v* from 30% to 50% of *S*, the jumping height varied from 6 (Movie S4) to 2 BL (Movie S5, Supporting Information). Durability tests at *v* = 30%*S* confirmed the BATE jumper's ability to sustain 100 consecutive jumps without notable performance degradation (Figure S7, Supporting information). Figure 3I details the air pressures involved, with *P*_ast_ (pressure from standby to activation, cyan bars) declining from ≈100 to ≈80 kPa, indicating lower activation energy required for higher *v* values. *P*_att_ (pressure at the triggered state, red bars) remained almost constant, highlighting stable operation in triggered conditions. The additional pressure needed for triggering *P*_tr_ decreased from ≈20 to ≈8 kPa (green dash and yellow bar), reflecting the reduced energy barrier for activation. Figure 3J exhibits the energy storage time required to trigger BATE jumper (the time between starting inflation and critical point, red dashed line), which declined from ≈190 to ≈110 ms as *v* increased from 30% to 50% of *S*. The green curve in the figure represents the take‐off velocity of BATE jumper, which declined from ≈2 (66.7 BL/s) to ≈0.8 m s^−1^ (26 BL/s), suggesting a trade‐off between jump height and activation speed.

Next, we examined how different beam lengths, *S*, influence BATE jumper's jumping performance for the same external frame (see the inset of Figure 3K). As shown in Figure 3K, with *S* increasing from 21 to 33 mm, the jumping height increased from 0 (the buckling beam of 21 mm length exhibited simple stability) to ≈100 mm (34 BL). The corresponding internal air pressure of the system, *P*_tr_, increased (with an increasing slope of 1.17) up to a maximum of 14 kPa (Figure 3L). Additionally, the energy storage time (Figure 3M) increased from ≈104 to ≈150 ms (with a slope of 3.83), while its take‐off velocity increased from 0.8 (26 BL s^−1^) to 1.1 m s^−1^ (36 BL s^−1^).

Thus, with different bistable buckling configurations, we can design jumping robots with various surmountable energy‐barriers and jumping performances. Moreover, it is noteworthy that this study mainly investigates the effect of different configurations, and we can further improve the jumping performance of the BATE jumper by reducing robot weight (see Supplementary Text 3, Figure S8, S9, Supporting information) or increasing the width of the spring steel beams (which would not increase the weight of frame to the same extent).

### 2.3 Turning from the Height‐Jump Mode to the Distance‐Jump Mode

**Figure 4:**
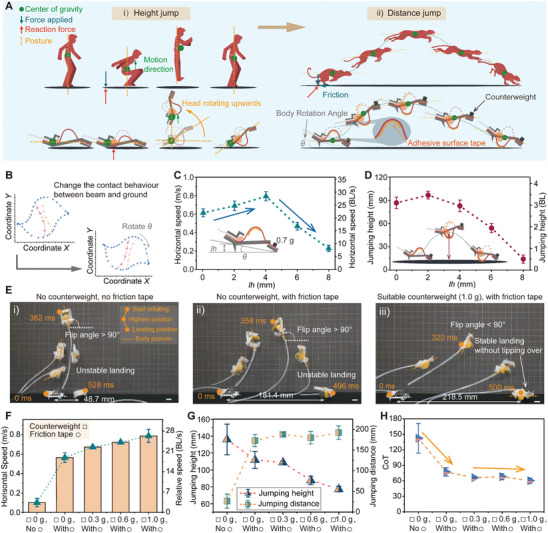
Switching of jumping modes and distance‐jump performances. A) Schematic showing comparative analysis of jumping mechanisms and postures of human height‐jump, cheetah distance‐jump, and prototype robots. B) Trajectory of two marked points during the snapping process captured by a high‐speed camera at 2000 fps. C,D) Variation of the horizontal velocity and jumping height of robot as a function of hind leg height, *lh* ranging from 0 to 8 mm. E) One‐cycle jumping motion of the BATE jumper snapshots for 3 scenarios. The 3 yellow markers denote the initial, the highest, and the landing position. F,G, and H) Variation of the forward jumping speed, jumping distance/height, and CoT of the robot as a function of friction tape and counterweight. Scale bar, 10 mm.

After demonstrating the excellent height‐jumping performance of the BATE jumper, we observed that, although the jumper achieved greater heights at a vertical offset *v* of 30% of *S* compared to 50% of *S*, it covered a shorter horizontal distance upon landing. This phenomenon can be attributed to three main factors (**Figure** **4**A‐i): first, the direction of the force generated when the bistable beam undergoes snapping in contact with the ground (e.g., only upward reaction forces can theoretically be generated in the symmetrical configuration), lacking the horizontal component necessary for forward propulsion; then, the smooth surface of the spring steel beam limits the generation of enough friction for the forward movement; finally, the center of gravity of the BATE jumper is always at the tail owing to the airbag actuator and air tubes, so the whole body undergoes a rotation of ≈90° (yellow arrow) during the jumping process until the head and tail are aligned vertically (Movie S6, Supporting Information), akin to a human high jump in situ.^[^ ^42^ ^]^

Figure 4A‐ii illustrates the process of a cheetah jumping forward and the superior distance‐jumping performance mainly originates from two factors^[^ ^43^, ^44^, ^45^ ^]^: first, contact with the ground generates a sufficiently large reaction force forward and upward (forward friction and vertical upward ground reaction force); second, the center of gravity at the waist can guarantee the stability of the whole jumping process. Therefore, we first rotated the body by a certain angle θ (by adding the rear leg design), altering ground contact dynamics (e.g., the location, duration, and direction) to boost both upward and forward forces. Further, we added a counterweight at the head to balance the center of gravity, mirroring the stability provided by a cheetah's waist, and applied a layer of friction tape (double‐sided polyimide adhesive with a thick of 50 µm) to the beam surface to increase the friction with the ground.

We captured the bistable beam's snapping motion (at *v* = 30*%S*, 2000 frames per second, see Figure S10 and Movie S7, Supporting information), with Figure 4B detailing the trajectories of marked points between two equilibrium states. Rotating the robot's body by angle θ alters beam‐ground contact, which could enhance the jumping performance. For simplicity, we replaced θ with the hind leg height *lh* (ranging from 0 to 7 mm, corresponding to 0 to 20° for θ). With a consistent 0.7 g head counterweight and friction tape in place, Figure 4C shows that as *lh* increased, the BATE jumper's horizontal velocity rose slowly, peaking at 28.3 BL s^−1^ (a 28.6% improvement) at *lh* = 4 mm and dropped to 8 BL s^−1^ at *lh* = 8 mm. The jumping heights, depicted in Figure 4D, reached a maximum of 3.4 BL at *lh* = 2 mm. Note that for *lh* values greater than 4 mm, the buckling beams’ insufficient impact with the ground resulted in reduced jumping velocities and heights.

To evaluate the effect of counterweight and friction on BATE jumper's jumping performance, Figure 4E delineates three scenarios: i) without counterweight and friction tape, the jumper deflected vertically due to the air tube's drag and the center of gravity located at the tail, achieving a jumping height of 5.5 BL but limited distance less than 2 BL (Movie S8, Supporting Information); ii) the introduction of friction tape markedly augmented the forward jumping distance to over 180 mm (6 BL); however, the jumper's posture was very unstable and flipped over 90°; iii) the subsequent addition of a 1.0 g counterweight not only extended the forward jumping distance to beyond 210 mm (7.5 BL) but also improved the overall stability during the jump, ensuring a landing without the body tipping over (Movie S9, Supporting Information).

Figures 4F,G illustrate that the application of friction tape significantly boosted the robot's horizontal velocity from 3.5 to 20 BL s^−1^ (a 4.7‐fold improvement) and its jumping distance from 0.9 to 5.8 BL (a 5.4‐fold improvement), which then experienced a gradual rise with increasing counterweights due to the trade‐off between increased horizontal jumping speed and reduced jumping height. Finally, we used cost of transport (CoT = *E*_in_/(*m* × *g* × *d*)) to evaluate the energy efficiency of the BATE jumper (Figure 4H), where *E*_in_ is the energy input, *m* is the mass, *g* is the standard gravity, and *d* the combined jumping height and distance. The energy input to the system originates from the electrical supply of the pneumatic pump (5 V, 0.76 A). The lowest CoT (≈60) of the system was achieved with a counterweight of 0.1 g. Overall, by adding friction tape and adjusting counterweights, we improved the jumping stability of the BATE jumper with a trade‐off of a 44% loss in jumping height for a more than 6‐fold increase in horizontal velocity and jumping distance, along with a 60% reduction in CoT.

### 2.4 Continuous Jump and Demonstrations

**Figure 5:**
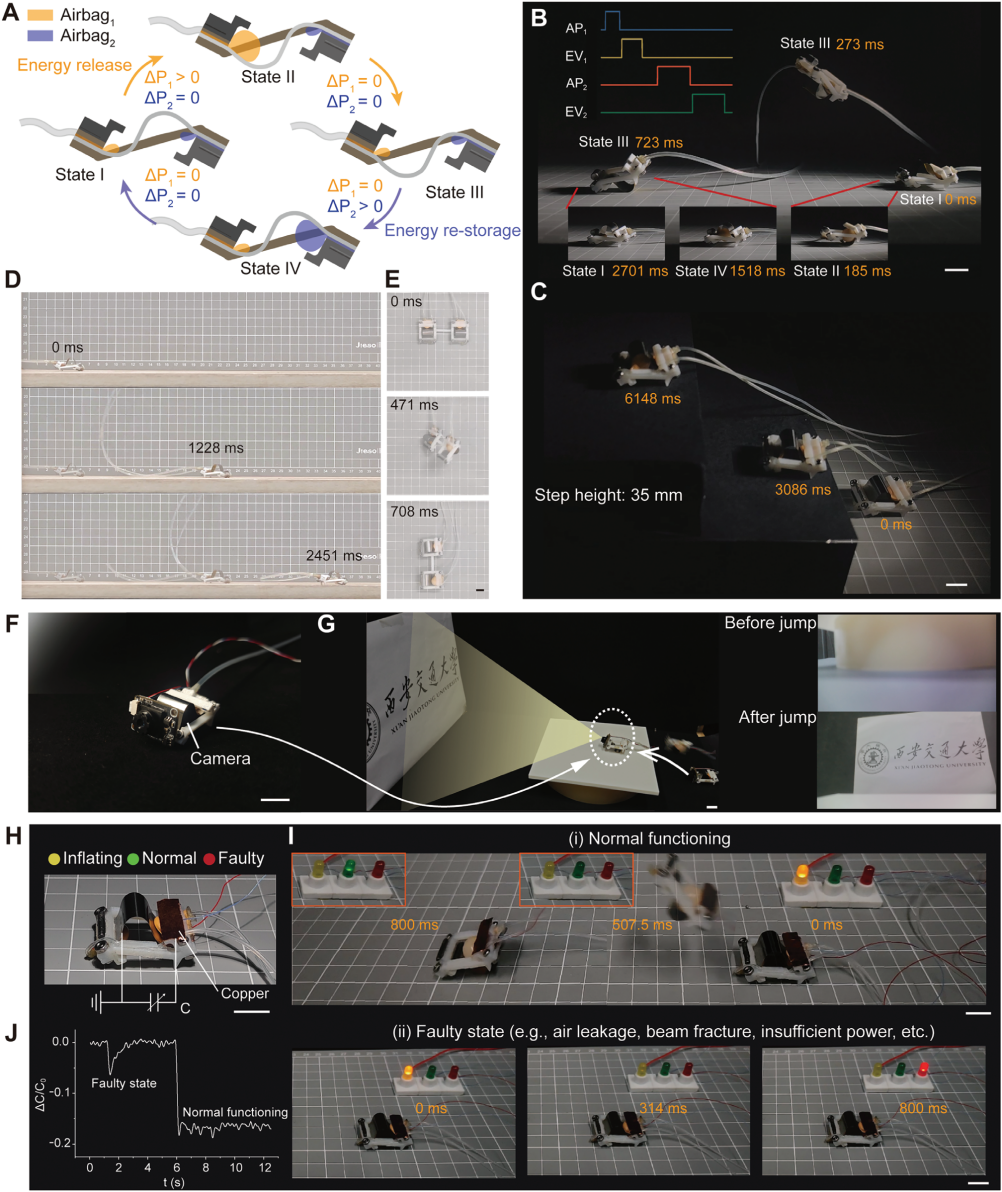
Robot's continuous jump steered jump and status detection. A) The BATE jumper's design features dual airbags for continuous jumping, delineated into four stages. B) Time‐lapse images capture the four stages in A consists of releasing energy (State I‐II‐III) and re‐storing energy (State III‐IV‐I). The inset details the sequential control of each component (air pump (AP), exhaust valve (EV), the numerical subscripts 1 and 2 correspond to the back/front airbags, respectively). C,D, and E) Robot's performance in continuous jumping on steps and flat ground (PVC (Polyvinyl chloride) substrate) as well as steering. F,G) The BATE jumper equipped with a camera to capture the target object. H) Design of the capacitive‐sensing‐based status monitoring system of the robot. I,J) Monitoring system with feedback indication through different colored LEDs and the relative change in capacitance signals during normal and faulty states. Scale bar, 10 mm.

To enable the BATE jumper to perform continuous jumping, we integrated a same airbag in its head that restores the energy of the bistable system (**Figure** **5**A). The BATE jumper undergoes a cyclical four‐phase motion: starting with airbag_1_ inflating to trigger the bistable mechanism; followed by airbag_1_ deflating, transitioning the system from state II to state III; the cycle continues with the airbag_2_ inflating and the bistable mechanism restores energy; concluding with airbag_2_ deflating, resetting the system to its original state. Figure 5B illustrates the video frames of the robot completing a cycle in 2071 ms with a jumping distance of 4.8 BL (Movie S10, Supporting Information). We also set up steps with a height of 35 mm, and the BATE jumper accomplished two successive jumps in 6148 ms (Figure 5C; Movie S11, Supporting Information). Figure 5D and Movie S12 (Supporting Information) depict the BATE jumper's continuous jumping process on a flat board, maintaining a velocity of 4.4 BL s^−1^. Additionally, we assembled a dual‐body BATE jumper laterally to achieve a turning speed of 127° s^−1^ (Figure 5E; Movie S13, Supporting Information). The BATE jumper is designed to carry functional devices like cameras and temperature sensors, broadening its detection and monitoring capabilities. Here, we demonstrate installing an 18 mm × 12 mm × 7 mm camera on the BATE jumper's head for target object detection. This setup enables the BATE jumper to overcome visual obstacles and survey areas inaccessible to crawling robots. The BATE jumper was also designed to carry functional devices such as cameras and temperature sensors, broadening its detection and monitoring capabilities (see Supplementary Text 5, Supporting information). As an example, we installed an 18 mm × 12 mm × 7 mm camera on the BATE jumper's head for target object detection (Figure 5F). The BATE jumper successfully overcame visual obstacles and surveyed areas inaccessible by crawling robots (Movie S14, Supporting Information; Figure 5G).

To enhance the BATE jumper's functionality and reliability, especially in closed environments, we affixed a copper foil (7 mm × 21 mm × 0.04 mm) on the back end of the BATE jumper, which forms a capacitive sensor with the spring steel beam, further adding self‐sensing capabilities to the original actuation element (Figure 5H; Movie S15, Supporting Information). An LCR (inductance, capacitance, and resistance) meter tracked the capacitance value (*C*) to ascertain the BATE jumper's motion status and detect malfunctions, indicated by LED (light‐emitting diode) colors (i.e., yellow means that the device is inflating, green means normal operation, and red means malfunction, as shown in Figure 5I). Figure 5J shows the relative change in *C* (Δ*C*/*C*_0_) when the BATE jumper is in normal and faulty (air leakage) states, respectively. The normal function yields Δ*C/C*_0_ = −0.18, whereas the air leakage results in Δ*C*/*C*_0_ = −0.07, a 2.6‐fold noticeable difference of the signal change between the two states.

## 3 Conclusion

In this study, we introduced an innovative insect‐scale jumping robot that leverages a novel offset‐buckling bistable design to achieve tunable energy barriers. This design significantly reduced the *F*_crit_ and *D*_crit_ required to trigger the snapping by up to six and nine times less, respectively, compared with the conventional symmetric buckling, improving the efficiency of energy release and restorage. Our BATE jumper, with its compact body size of just 15 mm, showcased the mode‐transitioning capacity (i.e., height/distance jump), achieving remarkable jumping performance up to 12.7 and 20 BL in different directions. The integration of dual airbags and a capacitive sensor allows BATE jumper for agile continuous jumping with energy release and re‐storage times of less than 300 ms and real‐time status detection. Future research will explore untethered operations for enhancing the robot's autonomy and develop a tunable adhesive friction tape for continuous transition between height‐ and distance‐jump modes. By integrating onboard power sources and control systems, the BATE jumper could operate independently, navigating and adapting to complex environments without external intervention.

## 4 Experimental Section

### Fabrication of BATE Jumper

The framework of the BATE jumper was 3D printed by a commercial printer (X1, Bambu Lab Co., Ltd, China) using PLA material. The beam was made of stainless steel (cross‐section 12.7 mm × 0.05 mm, XIDE Co., Ltd, China). The airbag was obtained by cutting a long latex balloon (Zhejiang Huachi Industry & Trade Co.) and retaining its head. It is connected by two kinds of thin tubes with outer diameter × inner diameter of 1 × 0.5 mm and 0.5 × 1 mm (weighting 22.9 and 10 mg cm^−1^). A notch on the rigid frame at the edge, which matches the outer diameter of the tube, constrains the position of the air tube, and balloon, and all components were assembled with nuts (nominal diameter, 1 mm) and bolts. The total cost of each BATE jumper was <5 RMB, and the time for fabrication and assembly was <30 min.

### Pneumatic Control

The pneumatic control system consists of four components, which were in charge of control, pneumatic setup, power supply, and feedback system (see Supplementary Text 6, Figures S11 and Figure S15, Supporting information). The control module consists of the computer and microcontroller (Mega 2560, Arduino Inc.) that send commands to a DFD (Dual full‐bridge driver, L298N, STMicroelectronics Inc.) module. The DFD module regulates the air pump (Koge Micro Tech. Co., China) and the solenoid (PIN YA Tech., China). The air pressure sensor (DP‐101/2, Panasonic Co., Ltd.) was connected to the air path, and the oscilloscope (4 Series MSO Mixed Signal Oscilloscope, TEKTRONIX, Inc.) captures the air pressure data in real‐time.

### Finite element Analysis

The commercial finite element software (ABAQUS, Dassault Systems Inc.) was used to perform the simulations (via static‐general analysis in Abaqus/Standard). The beam was modeled as a 2D beam cell with dimensions of 100 mm × 12.7 mm × 0.05 mm, density of 7850 kg m^−3^, and elastic properties with a Young's modulus of 194 GPa and a Poisson's ratio of 0.3. To implement boundary actuation, unidirectional displacement inputs were applied to two rigid bodies, which were located at a horizontal distance (10*%S*) from the left and right ends of buckling beams.

### Experimental Procedures and Motion Capture

A tensile testing machine (PT‐1176, POOTAB Co., Ltd.) was used to measure the force‐displacement relationship during the snapping for different buckling configurations. Robot take‐off and landing experiments were performed on a paper surface with a scaled PVC mat background (with 1 cm squares). An M120 camera (HF Agile Device Co.)was used to capture the ultra‐high‐speed video (the snapping motion) at 2000 fps.

## Conflict of Interest

The authors declare no conflict of interest.

## Supporting information

Supporting Information

Supplemental Movie 1

Supplemental Movie 2

Supplemental Movie 3

Supplemental Movie 4

Supplemental Movie 5

Supplemental Movie 6

Supplemental Movie 7

Supplemental Movie 8

Supplemental Movie 9

Supplemental Movie 10

Supplemental Movie 11

Supplemental Movie 12

Supplemental Movie 13

Supplemental Movie 14

Supplemental Movie 15

## Data Availability

The data that support the findings of this study are available from the corresponding author upon reasonable request.
