# Supplementary material for: Bistable Insect‐Scale Jumpers with Tunable Energy Barriers for Multimodal Locomotion
Source: Adv Sci (Weinh). 2024 Jul 7;11(34):2404404. doi: 10.1002/advs.202404404 (PMC11425846; doi:10.1002/advs.202404404)
Supplement: Supplementary file 1 — Supporting Information [file ADVS-11-2404404-s012.docx]

Supporting Information

**Bistable Insect-Scale Jumpers with Tunable Energy Barriers for Multimodal Locomotion**

*Qingkai Guo^1^, Yu Sun^1^*, Tianxiang Zhang^1^, Shiyu Xie^1^, Xuefeng Chen^1^, Zhuang Zhang^2,3^*, Hanqing Jiang^2,3,4^*, Laihao Yang^1^**

^1^School of Mechanical Engineering, Xi’an Jiaotong University, Xi’an 710049, China.

^2^School of Engineering, Westlake University, Hangzhou, Zhejiang 310030, China.

^3^Westlake Institute for Advanced Study, Hangzhou, Zhejiang 310024, China.

^4^Research Center for Industries of the Future, Westlake University, Hangzhou, Zhejiang 310030, China

*Correspondence to: Yu Sun (yu.sun@xjtu.edu.cn), Zhuang Zhang (zhangzhuang@westlake.edu.cn), Hanqing Jiang (hanqing.jiang@westlake.edu.cn) and Laihao Yang ([yanglaihao@xjtu.edu.cn](mailto:yanglaihao@xjtu.edu.cn))

**The PDF file includes:**

Supplementary Text 1 to Text 8

Figure S1 to S18

Table S1 to S4

Legends for Movies S1 to S15

References

**Other Supplementary Material for this manuscript includes the following:**

Movies S1 to S15

**Supplementary Text**

**1. Mechanisms behind low and high energy barrier sides in offset buckling structures**

We performed fixed-direction load input for both sides of a buckling beam with configuration parameters ([*h, v*] = [60, 50]*%S*) in finite-element and real-world scenarios, respectively, keeping the same material parameters and boundary conditions in both scenarios.

In the first scenario (see Figure S1A), where the bistable structure is actuated from the HES, the beam exhibits an up-convex local shape near the left side of the force application point, which opposes the downward direction of the applied force. This induces a pronounced down-concave localized deformation of the beam buckling when triggering the buckling beam, resulting in a large *F*_crit_ and *D*_crit_.

Conversely, in the second scenario (see Figure S1B), where the bistable structure is actuated from the LES, the beam exhibits a down-concave local shape near the right side of the force application point, which aligns with the direction of the applied force. This prevents the formation of a new downward localized large deformation with high curvature, leading to a small *F*_crit_ and *D*_crit_ for snap-through.

**2. Large displacement buckling behavior analysis**

In this section, we aim to determine the configuration of a buckling beam for different buckling parameters (i.e., the position of the clamped beam ends). By determining the number of inflection points, we can identify the stability state of the beam, either single-stable or bistable. The widely used Euler-Bernoulli theory (linearized, small-deflection theory or second-order theory) applies only for infinitely small deformations from an initial stressed state of the structure. Another common method is the approximate energy approach, which is more suitable for determining the initial nonlinear deflection behavior. In this work, the boundary conditions (i.e., the position coordinates of both ends, which are of fixed support) are given, and we will use the Elastica (elliptic integrals) to obtain an exact equilibrium solution for beam shapes.

Referring the pioneering works of Timoshenko and Hartono^[37–40]^, we consider a thin beam of bending stiffness *EI* under axial force *N* at both ends (as shown in Figure. S3A i,ii), the coordinate system is setting by one end of the beam). We assume that the length of the deflected centroidal axis of the column does not change, and ignore shear deformations. Differentiating with regard to *s* (Figure S3A iii), we obtain the differential equations as follows:

$$\begin{aligned} EI\frac{d^{2}\theta}{ds^{2}}=-N\sin\theta\#\left( S1a \right) \\ \frac{dy}{ds}=\sin\theta\#(S1b) \\ \frac{dx}{ds}=\cos\theta\#(S1c) \end{aligned}$$

with boundary conditions: d*θ*/d*s* = 0, when *θ* = *α*.

Where *s* is the length coordinate measured from the origin (the left end) along the deflection curve and *θ* the slope angle.

The solution is mathematically identical to the equation that describes large oscillations of a pendulum.

$$\begin{aligned} \frac{EI}{2N\left( \frac{d\theta}{ds} \right)^{2}}=\cos\theta-\cos\alpha\#\left( S2 \right) \end{aligned}$$

$\begin{aligned} \begin{aligned} &ds=\sqrt{\frac{EI}{2N}}\frac{d\theta}{\sqrt{\cos\theta-\cos\alpha}} \\ & =\frac{1}{2}\sqrt{\frac{EI}{N}}\frac{d\theta}{\sqrt{\sin^{2} \frac{\alpha}{2}-\sin^{2} \frac{\theta}{2}}} \end{aligned}\#\left( S3 \right) \end{aligned}$

The equation S3 (the relation between the slope and the length coordinate s) completely defines the buckling configurations. Then, by introducing another variable $\varphi$ , we get

$$\begin{aligned} \sin\frac{\theta}{2}=\sin\frac{\alpha}{2}\sin\varphi\#\left( S4 \right) \end{aligned}$$

It can be seen that when $\theta$ changes from 0 to $\alpha$ , $\sin\varphi$ changes from 0 to 1, and $\varphi$ changes from 0 to π/2. After differentiating (S4), we get

$$\begin{aligned} d\theta=\frac{2\sin\frac{\alpha}{2}\cos\varphi d\varphi}{\cos\frac{\theta}{2}}=\frac{2\sin\frac{\alpha}{2}\cos\varphi d\varphi}{\sqrt{1-\sin^{2} \frac{\alpha}{2}\sin^{2} \varphi}}\#\left( S5 \right) \end{aligned}$$

the large displacements of a buckling beam take the shape of the elastica, where the coordinates of any point ($x$ and $y$ ) and the length of the elastica $s$ are

$$\begin{aligned} s=\sqrt{\frac{EI}{N}}\left. \left[ K\left( \sin\frac{\alpha}{2} \right.,\frac{\pi}{2} \right)-K\left( \sin\frac{\alpha}{2} \right.,\left. \varphi\right) \right], \varphi=\sin^{-1} \left( \frac{\sin\frac{1}{2}\theta}{\sin\frac{1}{2}\alpha} \right) \#\left( S6a \right) \\ &x=\sqrt{\frac{EI}{N}}\left[ 2F\left( \sin\frac{1}{2}\alpha,\frac{1}{2}\pi\right)-2F\left( \sin\frac{1}{2}\alpha,\varphi\right)-K\left( \sin\frac{1}{2}\alpha,\frac{1}{2}\pi\right)+K\left( \sin\frac{1}{2}\alpha,\varphi\right) \right] \#\left( S6b \right) \\ &y=\sqrt{\frac{2\text{EI}}{N}\left( \cos\theta-\cos\alpha\right)}, -\alpha\leq\theta\leq\alpha\#\left( S6c \right) \end{aligned}$$

in which *K*($\sin\frac{1}{2}\alpha$,$\varphi$) and *F*($\sin\frac{1}{2}\alpha$,$\varphi$) are the elliptic integral of first and second kind, respectively (note that equations (S6a-c) are the same as equations (1a-c) of the manuscript). with

$$K(\sin\frac{1}{2}\left. \alpha,\varphi\right)=\int_{0}^{\varphi} \frac{d\theta}{\sqrt{1-\sin^{2} \frac{1}{2}\alpha\sin^{2} \theta}},F(\sin\frac{1}{2}\alpha,\varphi)=\int_{0}^{\varphi} \sqrt{1-\sin^{2} \frac{1}{2}\alpha\sin^{2} \theta}d\theta(S7)$$

For a single beam unit, we can get its elastica coordinates:

$$\begin{aligned} \boldsymbol{s}=\left[ s_{1},s_{2},\ldots,s_{n} \right]; \boldsymbol{x}=\left[ x_{1},x_{2},\ldots,x_{n} \right]; \boldsymbol{y} =\left[ y_{1},y_{2},\ldots,y_{n} \right]\boldsymbol{\#}\left( S8 \right) \end{aligned}$$

We get the shapes of the offset buckling beams by combining the elastica units and taking a part of them (Figs. S3B-C, note that we will focus on the elastica units with $\alpha$ less than 90° for ensuring smaller energy-barriers):

$$\begin{aligned} \boldsymbol{X}=\left[ \boldsymbol{x},\boldsymbol{x}+x_{n}*\boldsymbol{1},\boldsymbol{x}+2x_{n}*\boldsymbol{1} \right]; \boldsymbol{Y}=\left[ \boldsymbol{y,}-\boldsymbol{y,y} \right]\boldsymbol{;S}=\left[ \boldsymbol{s},\boldsymbol{s}+s_{n}*\boldsymbol{1},\boldsymbol{s}+2s_{n}*\boldsymbol{1} \right]\boldsymbol{\#}\left( S9 \right) \end{aligned}$$

Given a fixed total length *l* of the beam and an initial angle $\alpha$, we can determine the unique corresponding *N*, as well as the shapes of all eligible beams (including the end point coordinate (*x*_end_,*y*_end_)). Therefore, assuming that ns is index of the starting point (corresponding to the left end) of the beam, and using it as the coordinate origin, we rotate (clockwise) the shape coordinates until the tangent line at the starting point is horizontal to get the $\boldsymbol{X}^{\mathrm{rot}}\mathrm{and} \boldsymbol{Y}^{\mathrm{rot}}$.

For single-stable configurations with one inflection point, we need to satisfy the following conditions:

$$\begin{aligned} \boldsymbol{X}_{2n-\mathrm{ns}}^{\mathrm{rot}} -\boldsymbol{X}_{\mathrm{ns}}^{\mathrm{rot}} =x_{\mathrm{end}}; \boldsymbol{Y}_{2n-\mathrm{ns}}^{\mathrm{rot}} -\boldsymbol{Y}_{\mathrm{ns}}^{\mathrm{rot}} =y_{\mathrm{end}};\boldsymbol{S}_{2n-ns} -\boldsymbol{S}_{\mathrm{ns}} =l\#\left( S10 \right) \end{aligned}$$

While for bistable configurations with two inflection points, we have:

$$\begin{aligned} \boldsymbol{X}_{2n+\mathrm{ns}}^{\mathrm{rot}} -\boldsymbol{X}_{\mathrm{ns}}^{\mathrm{rot}} =x_{\mathrm{end}}; \boldsymbol{Y}_{2n+\mathrm{ns}}^{\mathrm{rot}} -\boldsymbol{Y}_{\mathrm{ns}}^{\mathrm{rot}} =y_{\mathrm{end}};\boldsymbol{S}_{2n+ns} -\boldsymbol{S}_{\mathrm{ns}} =l\#\left( S11 \right) \end{aligned}$$

**3. Effect of tubing tether and weight of robot body on jumper’s jumping performance.**

The tubing tethers affect the jumping performance of the BATE jumper in two ways: i) they increase the robot’s weight, lowering its maximum jump height. ii) they create line tension and sway during jumping, making the robot deviate from its desired direction, and this effect is more noticeable for lighter robots.

In measuring the height jump performance and distance jump performance of the BATE jumper (performance data shown in Figure 4 and Figure 5), we placed positioned it alongside silicone tubes (2 mm x 1 mm, weighing 22.9 mg/cm) on a platform. And the air tube that lifted off the ground accounted for approximately at most 10% of the BATE jumper’s weight during peak height measurements.

To evaluate the effect of tubing tethers and weight of robot body, we tested the jumper’s performance in three scenarios, maintaining the same beam configuration. We first placed tethers on the platform (Figure S8A); then suspended tethers (Figure S8B); and finally, while suspending tethers, we used a lighter body (a reduced fill rate PLA frame with Peek nuts weighting 1.4 g with a 25 mm BL, Figure S8C). Notably, with respect to first scenario, the jumper’s jumping height increased by 18.9% and 54% in the latter two scenarios, respectively.

**4.** **Comparison of the time efficiency of energy release and restorage in various of bistable robots.**

As illustrated in Figure S12 and Table S3, we have summarized recent developments in robots utilizing bistable mechanisms. Robots employing heat-actuated materials (e.g., Shape Memory Alloys (SMA) and Shape Memory Polymers (SMP)) exhibit long operating cycles more than 6 s due to their extended heat dissipation times. Additionally, robots using pneumatics achieve relatively short energy release and re-storage times but are significantly larger in size, often exceeding 6 cm in length. Our BATE jumper, designed with the offset-buckling configuration, offers substantial advantages in both size and the temporal efficiency of energy release and restorage. The compact size and rapid actuation capabilities of our BATE jumper make it highly suitable for insect-scale robotic applications.

**5. Evaluation of load capacity of the BATE jumper.**

When we were testing the jumping performance of the BATE jumper, we attached a counterweight (Figure S13A) to the head of the device by using glue (Cyanoacrylates). In a real-world scenario, we can extend the body of the BATE jumper by 3D printing it quickly. Further, we can attach cameras, temperature sensors, etc. on it (Figure S13B).

In Figure 4, we have investigated the effect of adding different counterweights on the jumping height and stability of the BATE jumper under the distance jump mode. To evaluate the load-bearing capacity of the BATE jumper, we tested the jump*ing* height at different loads and calculated the gravitational potential energy of the BATE jumper at its apex (refer to Figure S13C). The results show that the BATE jumper can achieve a jumping height of 50 mm with a 5 g load, twice its own weight. In addition, the energy expended for the BATE jumper’s jumping is approximately at around 4.2 mJ across different loads.

**6.** **Effect of power supply, and pressure feedback on the jumping performance of the BATE jumper.**

To evaluate the influence of the pneumatic system’s supply under varying voltages (ranging from 5 V to 7 V) on the BATE jumper’s performance, we tested it in height jump mode using the same configuration ([*h*, *v*] = [60, 30]%*S*) as in Figure 3. The result (see Figure S14) revealed that augmenting the voltage supplied to the air pump (i.e., enhancing the inflation rate), significantly reduced the energy storage time required to initiate the bistable beam's snap-through transition, from 185 ms at 5 V to approximately 140 ms at 7 V, a 22% reduction. However, the jumping height of the BATE jumper at different voltages maintained approximately same, as 195 mm. The reason for this phenomenon is that the energy for the BATE jumper’s jumping comes from the snap-through (i.e., energy release) of the bistable beam, whereas the role of the airbag is only limited to initiating the snap-through transition. Since the buckling beams' structure remains unchanged, the energy released during snap-through is uniform, resulting in consistent jumping height.

The feedback system for controlling bistable jumper actuation is crucial due to slight variations in component characteristics and assembly processes among different BATE jumpers, affecting the energy storage time. If the energy storage time is set too short, the bistable beam may fail to undergo snap-through; if too long, the airbag risks over-inflation, leading to energy wastage and potential airbag rupture (as illustrated in Figure S15A). Additionally, in the continuous jumping mode, untimely contraction of the airbag reduces the time efficiency of energy release and restorage. The airbag actuation during the height jump involves three stages: (stage I) initiation of the air pump, leading to gradual pressure increase; (stage II) reaching the critical point of the bistable beam, peaking internal pressure; and (stage III) snap-through transition, causing a sudden pressure drop.

Experimental comparisons of the BATE jumper with and without feedback (Figure S15B) revealed that, given the identical configuration of the buckling beams, the critical air pressure and time required to reach the critical point (i.e., energy barrier) were consistent across tests. Throughout the actuating process, the feedback system continuously monitors real-time changes in air pressure within the airbag and relays this information to the computer controller. In the presence of feedback, the system detected a sharp drop in air pressure following the critical point (Figure S15C), then triggered the exhaust valve to open and caused the airbag to contract to its initial state within 40 ms. Conversely, in the absence of feedback, the airbag continued to expand beyond the critical point, leading to a continuous increase in internal pressure. Performance tests, measured in terms of jumping height (Figure S15D,E), indicated that the jumping height of the BATE jumper, both with and without the feedback system, remained consistent at approximately 195 mm, owing to the same configuration of the buckling beam used (Figure S15F).

**7.** **Material selection for the fabrication and actuation of the BATE jumper.**

The characteristic length of our jumper ranges from 14 mm to 28 mm, with an irregular framework ideal for 3D printing. To support a buckling beam undergoing significant deformation, rigid materials are required. We evaluated three materials: PLA and PC, both printed via 3D FDM (Fused Deposition Modeling), and BLACK V4 resin, printed using 3D MSLA (Masked Stereo Lithography Apparatus). The comparison of these materials in terms of quality, working temperature, price, preparation time, and biodegradability is summarized in the Table S4. We found that the body frameworks printed in PLA, PC, and resin have similar weights but differ significantly in operating temperature limits. The PLA-based body deforms severely in environments exceeding 60 °C. In contrast, bodies made of PC and resin can operate in environments up to 120 °C. As shown in Figure S16, when subjected to 70 °C in a thermostat, the PLA-based body deformed severely within 10 minutes, whereas the PC-based and resin-based body remained intact at 120 °C after 60 minutes. However, PC and resin are much more expensive to produce, costing 3 and 11 times more than PLA, respectively. Additionally, 3D printing with resin takes 6.75 times longer than with PC and PLA. Of the three materials, only PLA is biodegradable, making it the more environmentally friendly option.

Overall, given that our testing environment is primarily at room temperature, PLA offers clear advantages in terms of cost, printing speed, and biodegradability. However, using PC material for the body allows the robot to operate in environments with temperatures between 60 °C and 160 °C.

In this work, we integrated the airbag into our offset-buckling bistable structure, which transforms the gradual deformation of the airbag into a rapid energy release, enabling motions such as jumping that require instantaneous energy release. We chose pneumatic airbags because actuating bistable structures requires materials capable of generating sufficient force and displacement to overcome energy barriers. We compared Dielectric Elastomer Actuators (DEA), Shape Memory Alloys (SMA), electromagnetic solutions, and pneumatic systems. While DEAs are suitable for high-frequency movements, their limited force/displacement output, complex fabrication, and pre-stretching requirements make them less viable for insect-scale jumping applications. Electromagnetic actuators face similar force output limitations at small scales. SMAs, despite their potential, are unsuitable due to slow thermal cycles incompatible with rapid actuation.

In contrast, our miniature airbag, measuring 8 mm by 8 mm in its deflated state and expanding to 16 mm within 200 ms (Figure S17A), exhibits remarkable expansion capabilities. Importantly, it achieves a blocking force of 4.8 N at a 10 mm stroke (Figure S17B), significantly outperforming other soft actuator materials in output force and displacement, thus meeting the criteria for our offset-buckling structure with an output force threshold greater than 0.4 N and displacement exceeding 5 mm. This positions our airbag as a superior soft actuating material for triggering the bistable structure.

**8.** **Retrieval and maintenance advantages of tethered robots in confined spaces.**

In real-world scenarios, insect-scale robots often malfunction in confined spaces that are difficult for humans to reach, making their retrieval challenging. However, air-tube-connected robots, like the BATE jumper, offer a practical advantage. If the robot gets trapped or malfunctions, it can be quickly retrieved by pulling on the air tubes, ensuring continuous operation and ease of maintenance (see Figure S18).

**
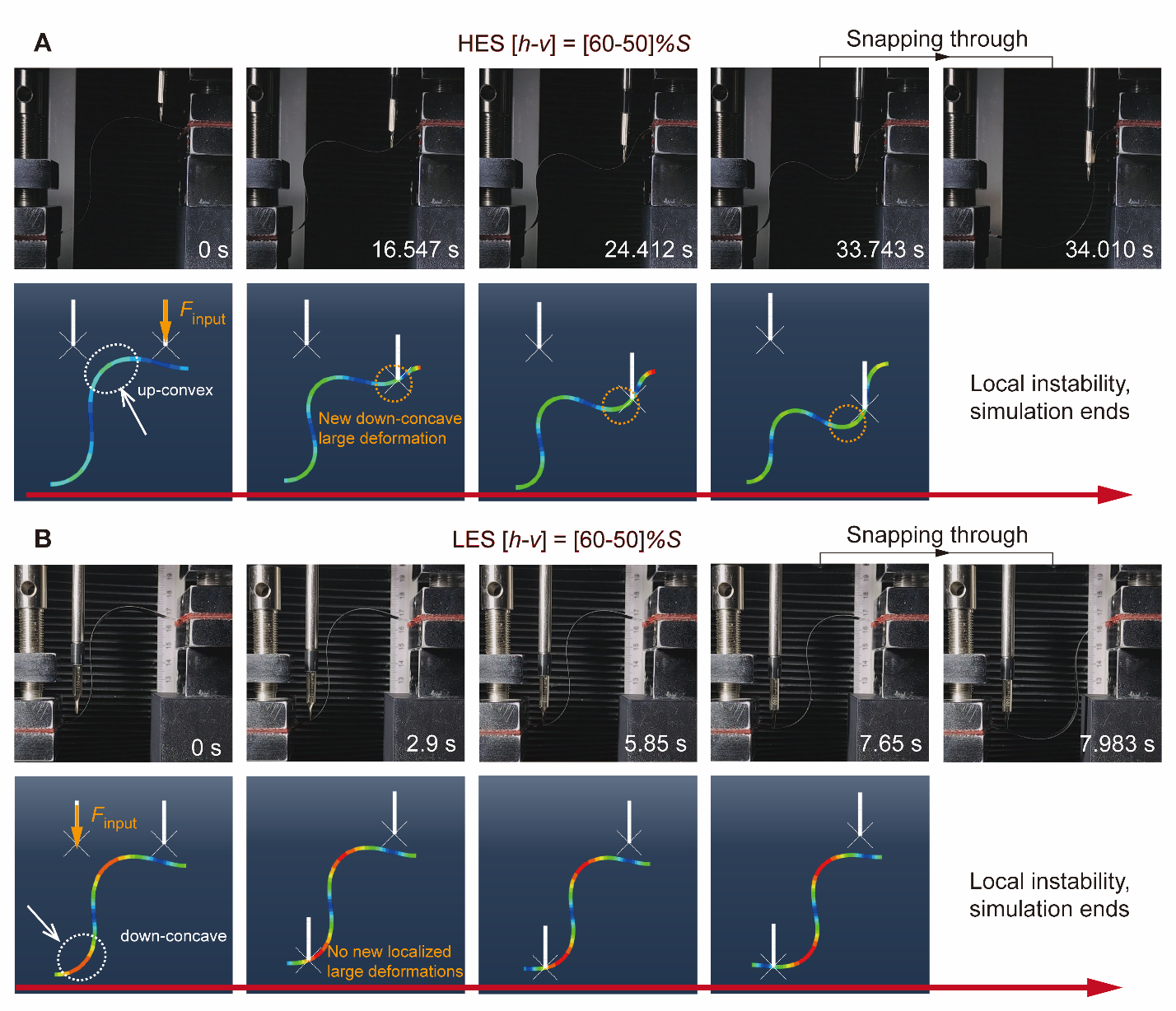
**

**Figure S1.** Shape changes procedure (of both experimental and simulated results) of buckling beams actuated from the HES and LES, respectively, in the offset buckling configuration ([*h, v*] = [60, 50]*%S*).


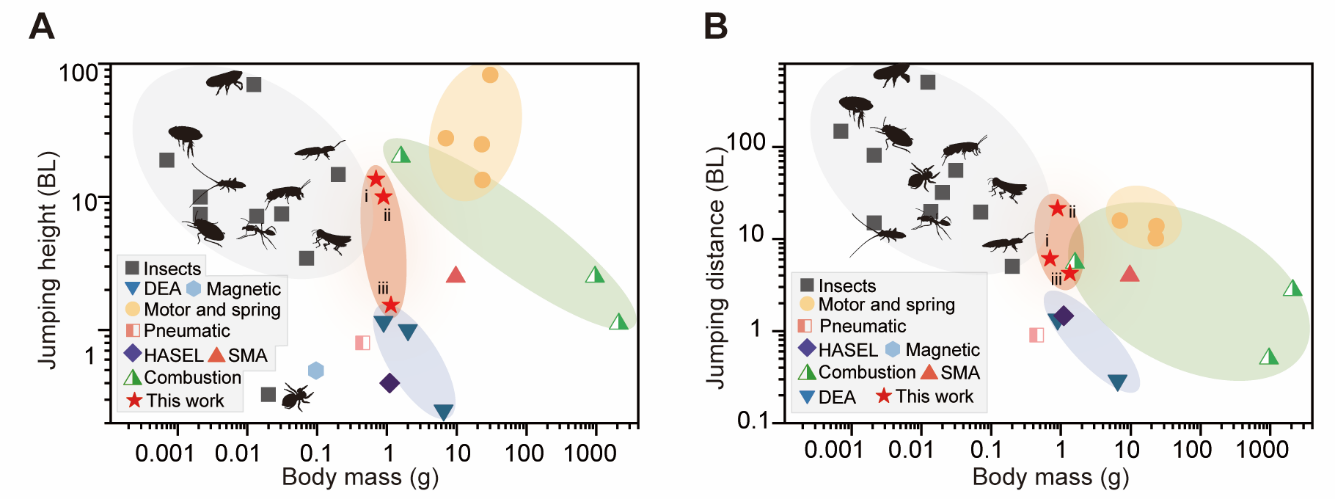


**Figure S2.** Comparison of jumping robots and various insects. **A**) The jumping heights/**B**) distances and body mass of this work, various insects and previous robots reported in the literatures^[9, 17, 25, 29, 34, 45–65]^.


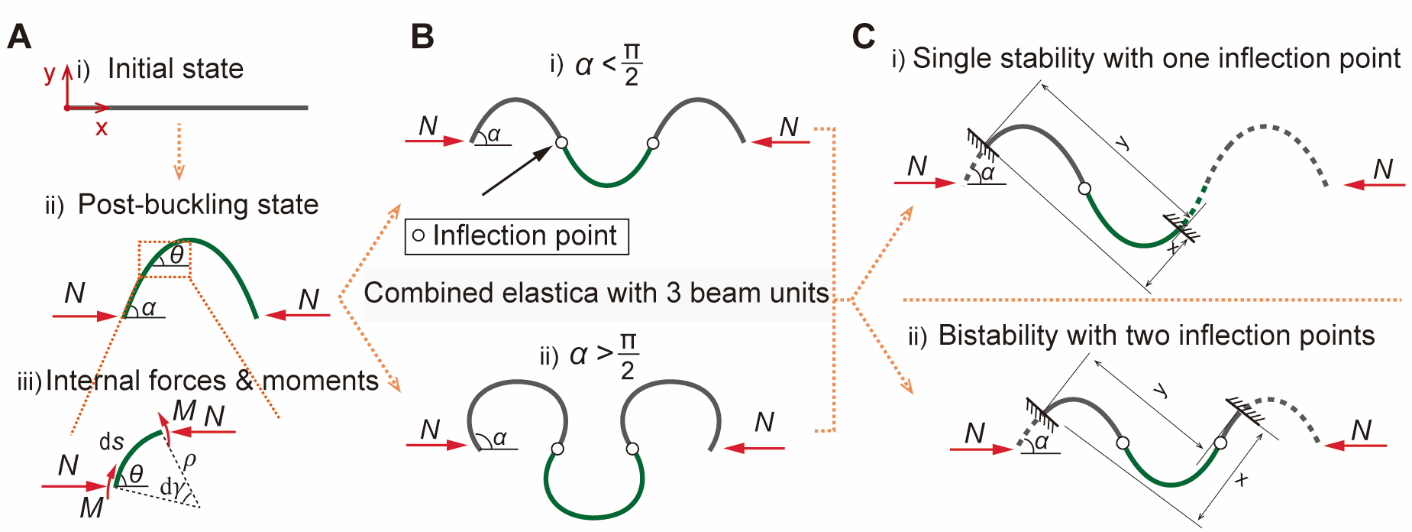


**Figure S3.** Large displacement behavior of the buckling beam. **A**) Internal forces and moments of beam unit (i-ii) and element (iii). **B**) Combined elastica with 3 beam units. Note that when $\boldsymbol{\alpha}$ > 90°, the beam buckles heavily and forms a large energy barrier, so we focus only on scenarios where $\boldsymbol{\alpha}$ < 90°. **C**) Simple stable and bistable configurations from the combined elastica.


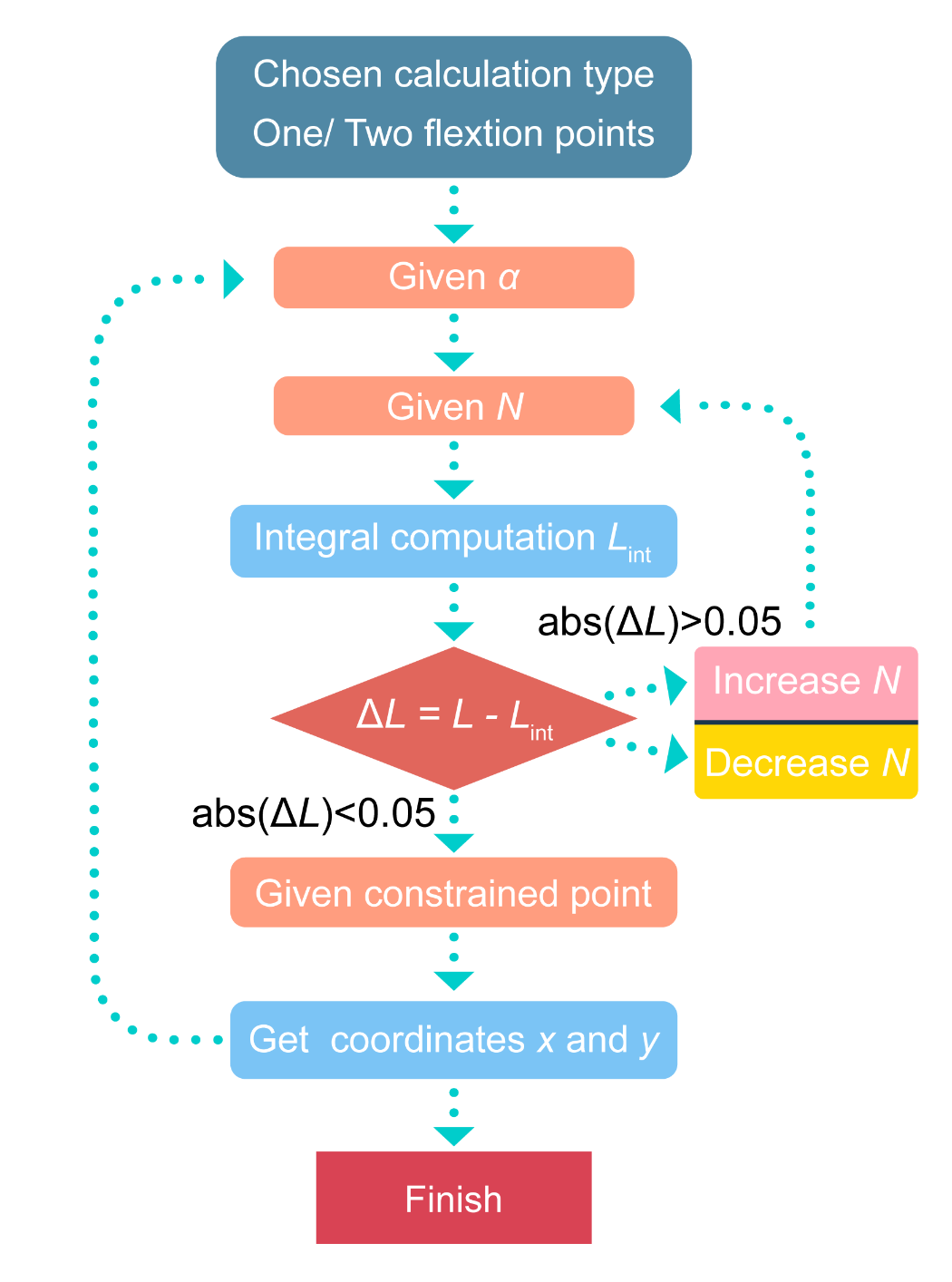


**Figure S4.** Flowchart of the analysis routine. It illustrates the analysis routine implemented in MATLAB to calculate the curved beam configurations with different buckling parameters.


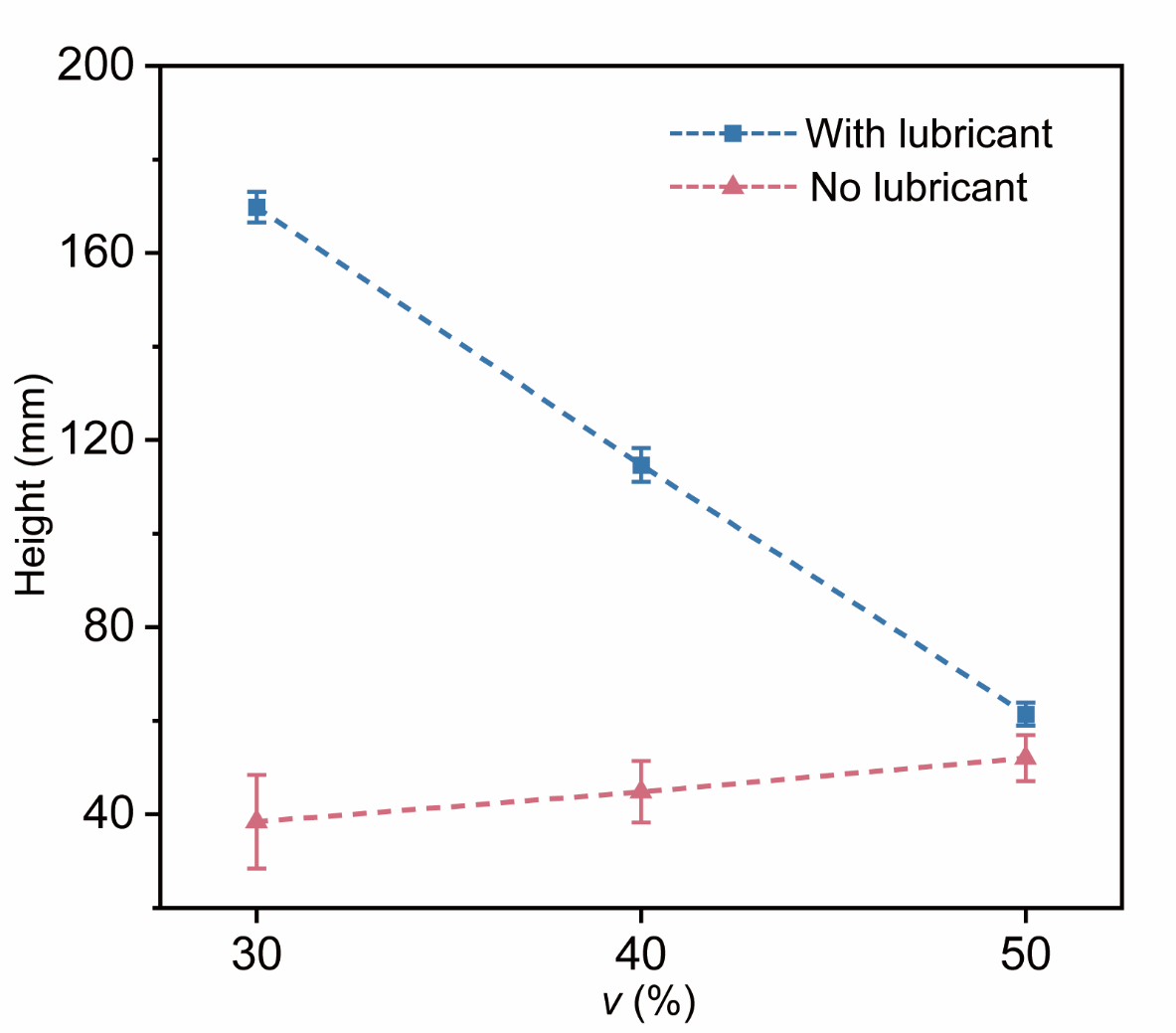


**Figure S5.** Effect of lubricant on the jumping height of BATE jumper.

The blue and red curves in Figure 3H represent the performance of the BATE jumper with and without lubricant, respectively. The result demonstrates that the lubricant improves the performance of the BATE jumper by lowering the friction during the snap-through process. As the energy barrier decreases, the lubricant effect becomes less significant due to the reduced friction between the miniature airbag actuator and the buckling beam.

**
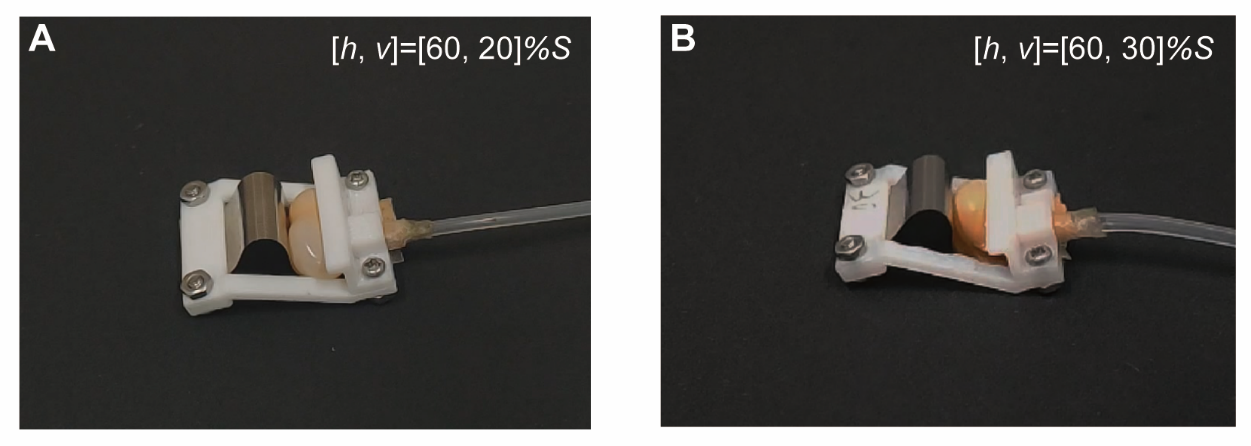
**

**Figure S6.** Expansion of the miniature airbag in configurations with different energy barriers. The configuration of BATE jumpers in **A**) was unable to be actuated with [*h, v*] = [60, 20]*%S* and in **B**) could be actuated with [*h, v*] = [60, 30]*%S*. The airbag’s expansion in unintended directions resulted in the failure of energy release from the snaping process (e.g., insufficient stroke to trigger snap-through, airbag explosion).

**
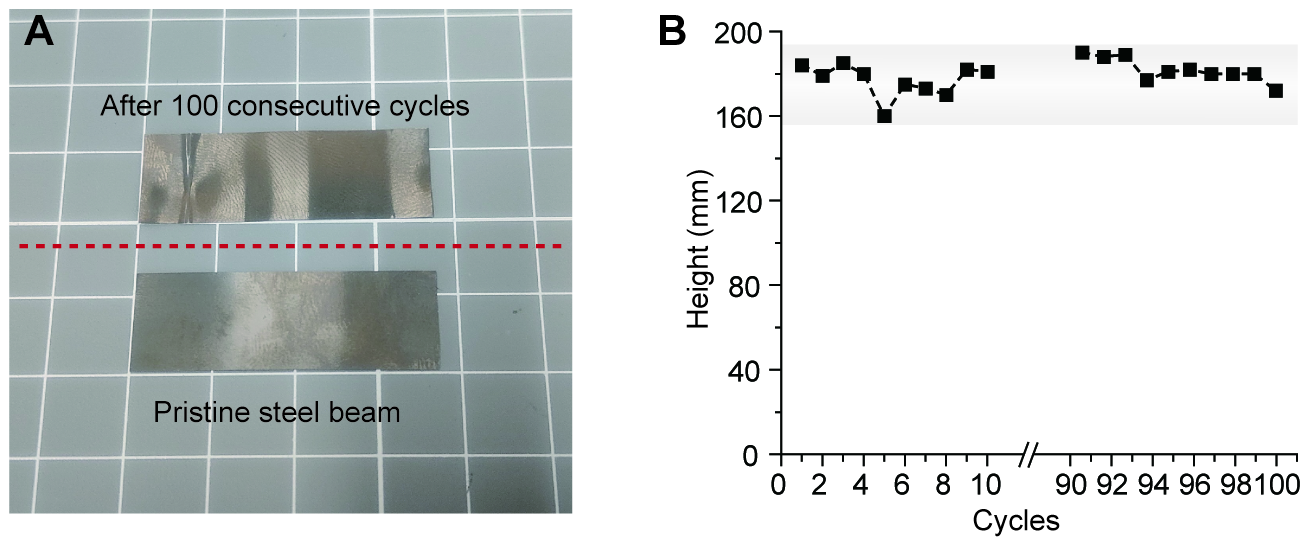
**

**Figure S7.** Durability test. We performed 100 consecutive jumping cycle tests on the configuration ([*h, v*] = [60, 30]*%S*), monitoring the spring steel beams’ fatigue and the BATE jumper’s height-jump performance variations. In **A**), we detected minor plastic deformations of the steel beam at the connection area with the frame. In **B**), we found that these deformations had a negligible impact on the height-jump performance after the 100 consecutive jumping cycles test.


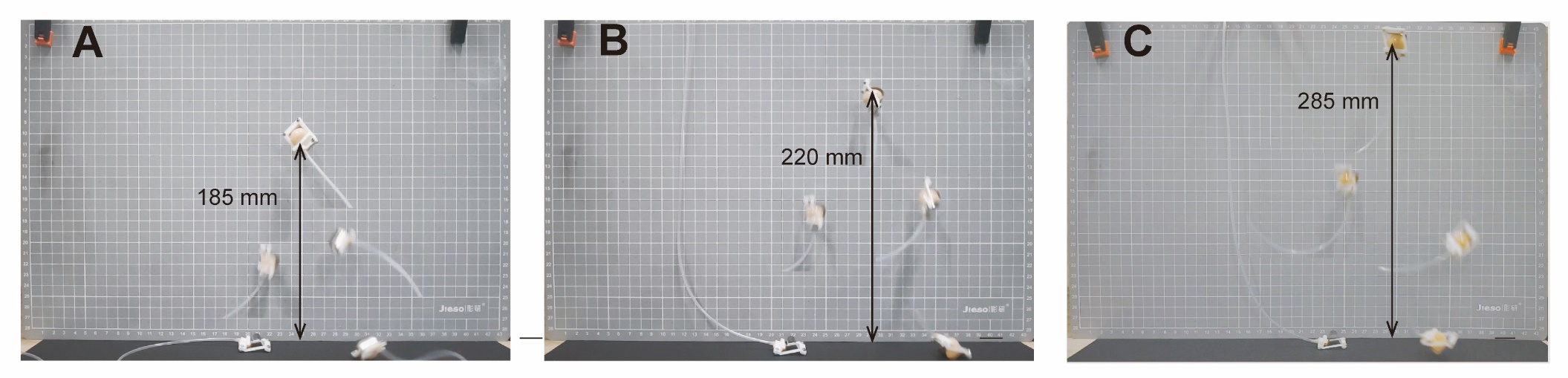


**Figure S8.** Jumping performance tested on three different scenarios in terms of tubing tether and weight of robot body. **A**) with tethers on the platform, **B**) tethers suspended, and **C**) tethers suspended with a lighter body (a reduced fill rate PLA frame with Peek nuts weighting 1.4 g with a 25 mm BL), maintaining the same beam configuration.

**
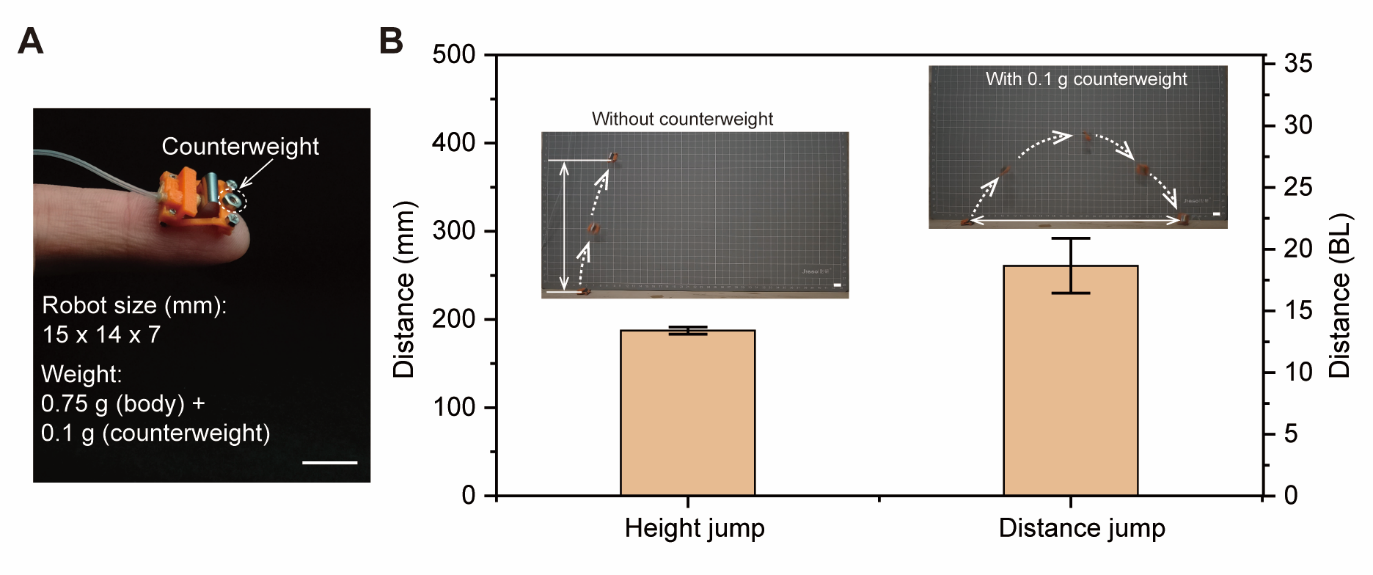
**

**Figure S9. Further miniaturization of the BATE jumper’s size.** (**A**) To further miniaturize the robot, the characteristic length of the BATE jumper (with [*h, v*] = [40, 30]*%S*) discussed in Figure 4 was halved to 15 mm, with a minimum body weight of 0.75 g (0.1 g counterweight was added to the head in distance-jump mode). Utilizing 30 µm thick, 9 mm wide steel sheets and lighter silicone tubing (1 mm x 0.5 mm, 10 mg/cm), this lighter and smaller tubing tether has reduced impact on the jumper’s performance. (**B**) shows the jumper’s performance at an average vertical jumping distance of 187.4 mm (12.5 BL) and an average horizontal jumping distance 261 mm (17.4 BL), as well as video frames of the jumper’s jumping process. Scale bar, 10 mm.

**
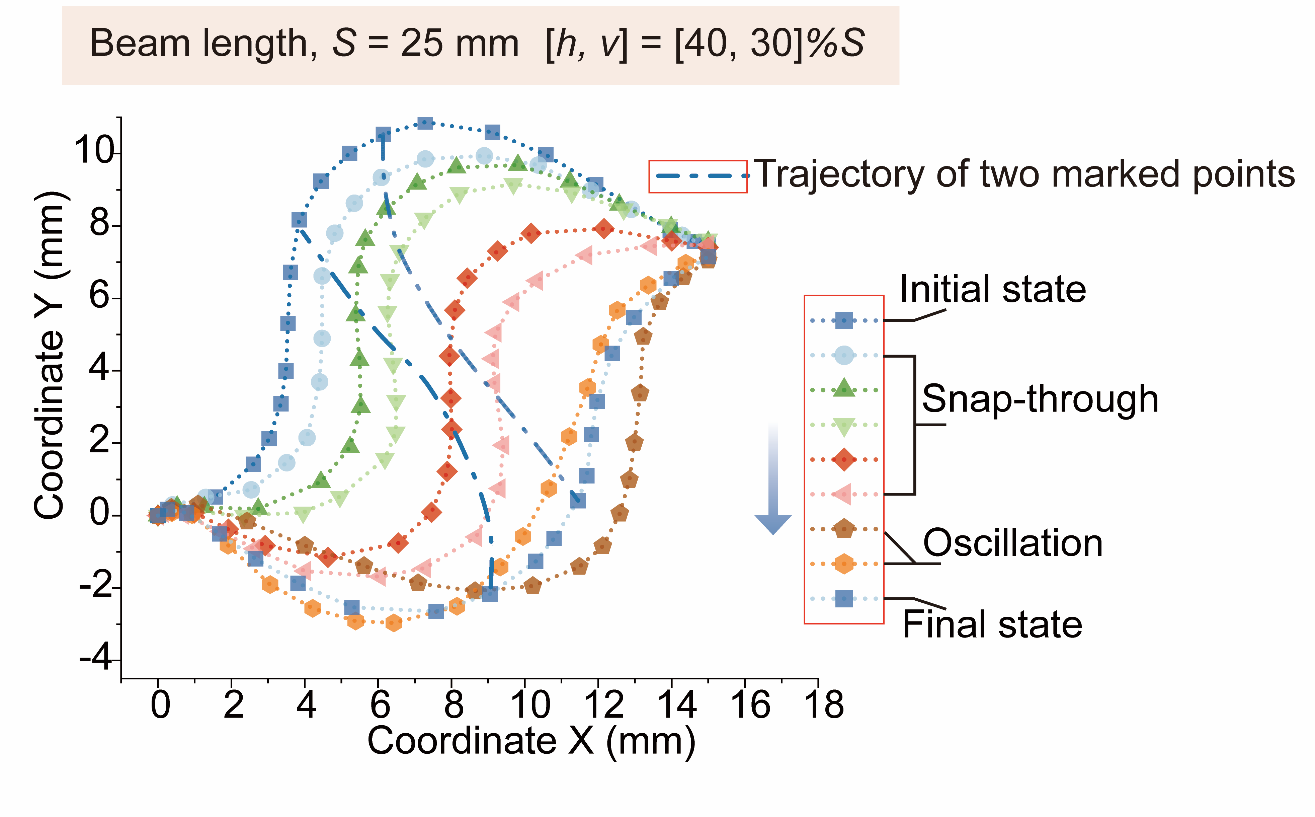
**

**Figure S10. Snapping dynamic process between two stable states captured by high-speed camera at 2000 fps.**


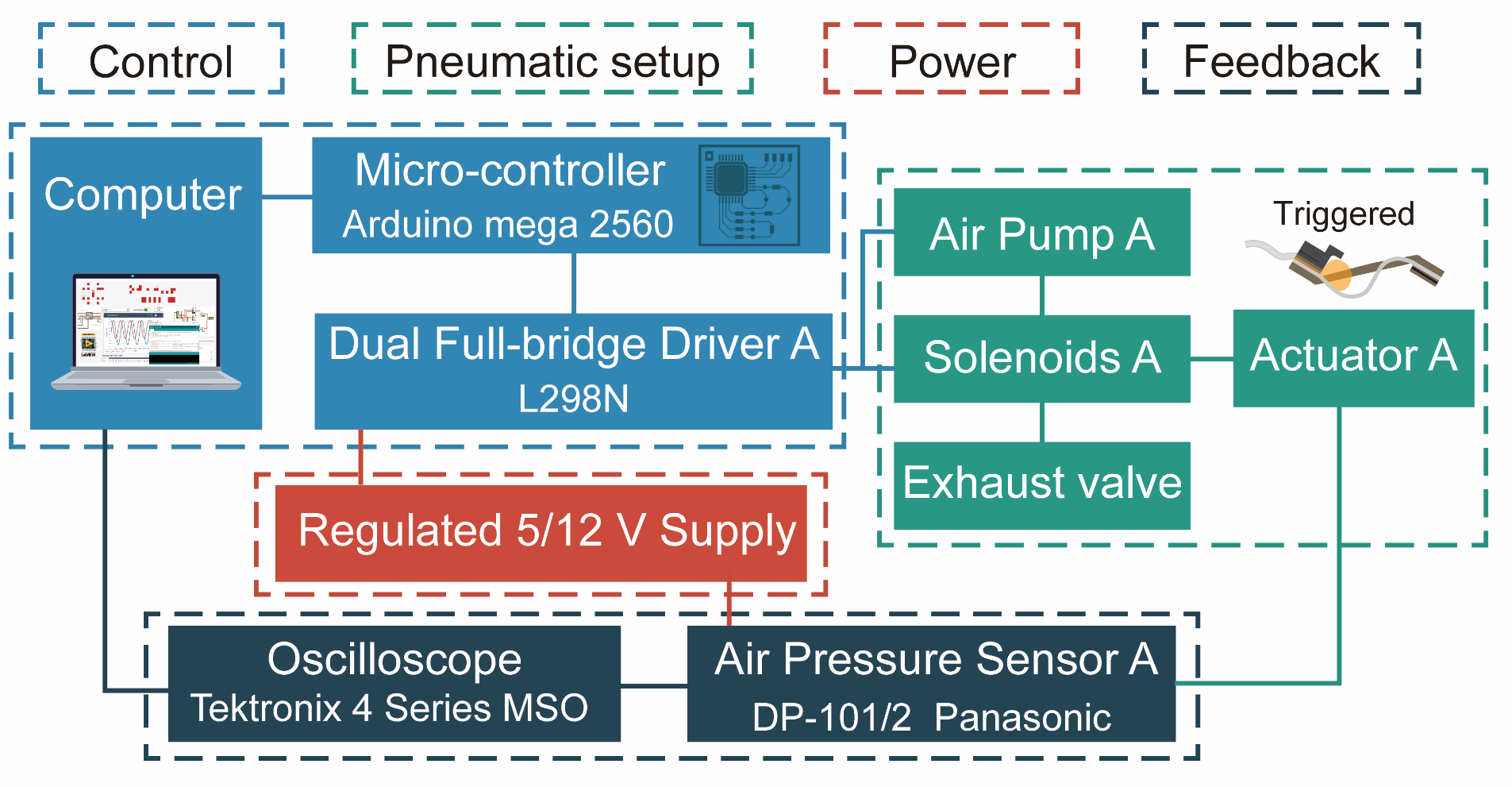


**Figure S11. Pneumatic control system for BATE jumper for single embedded actuator.** The whole system consists of four main parts: control components, pneumatic setup, power supply and feedback system.


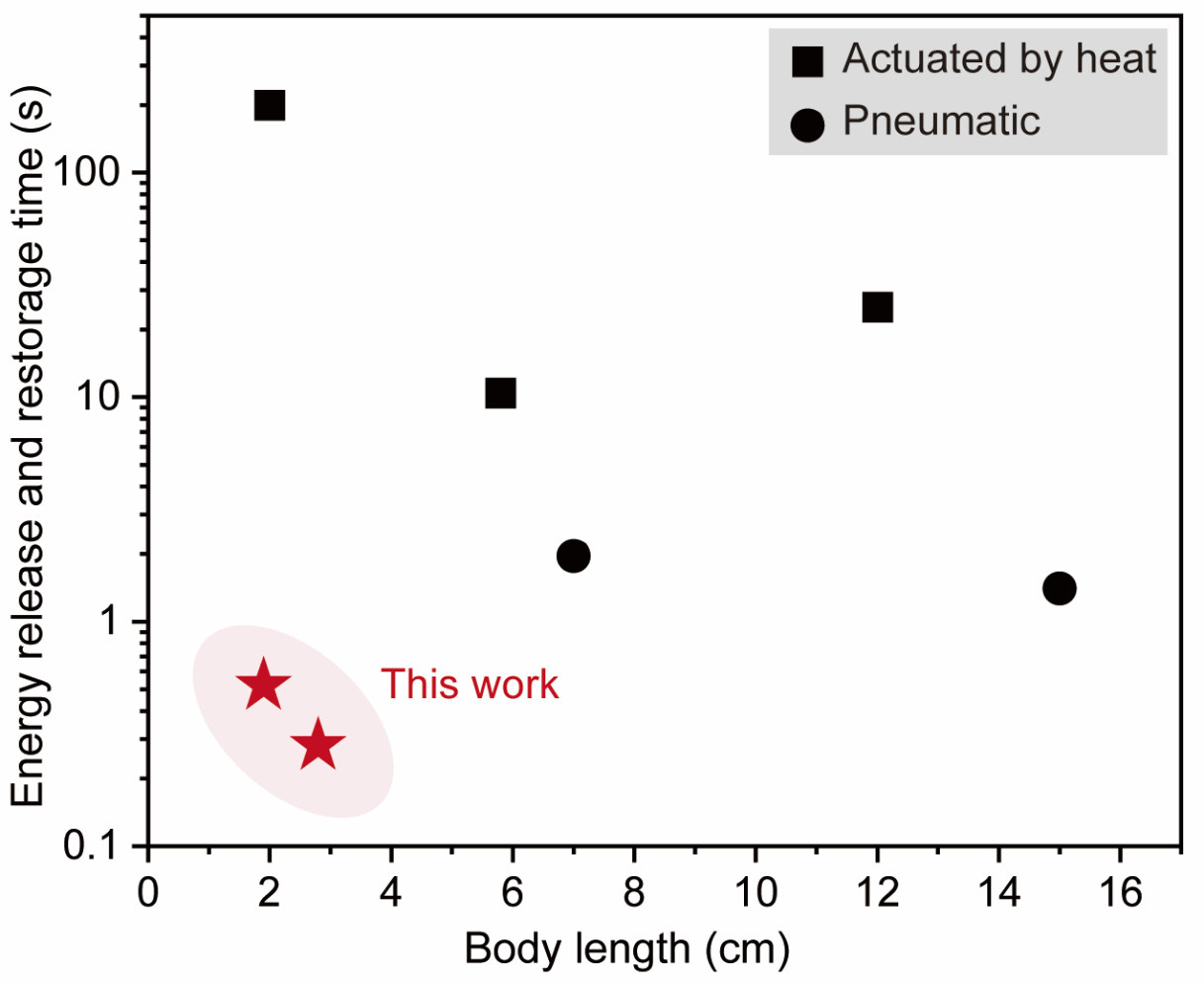


**Figure S12. Comparison of energy release and restorage times in bistable robots with different energy sources.**


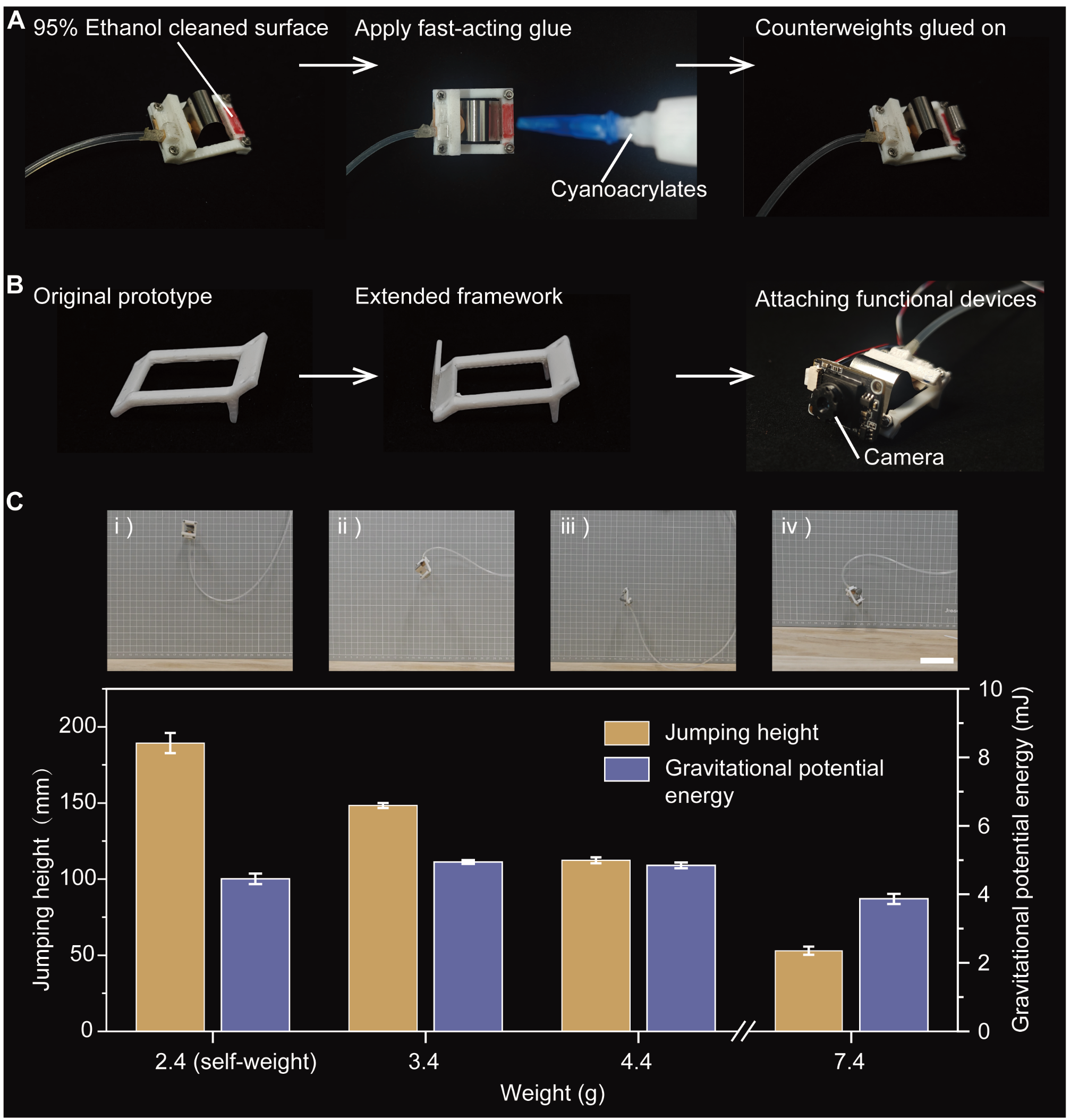


**Figure S13. A**) Process of attaching a counterweight to BATE jumpers. **B**) Body framework extensions design for attaching functional devices. **C)** Jumping height of BATE jumpers under different loads.


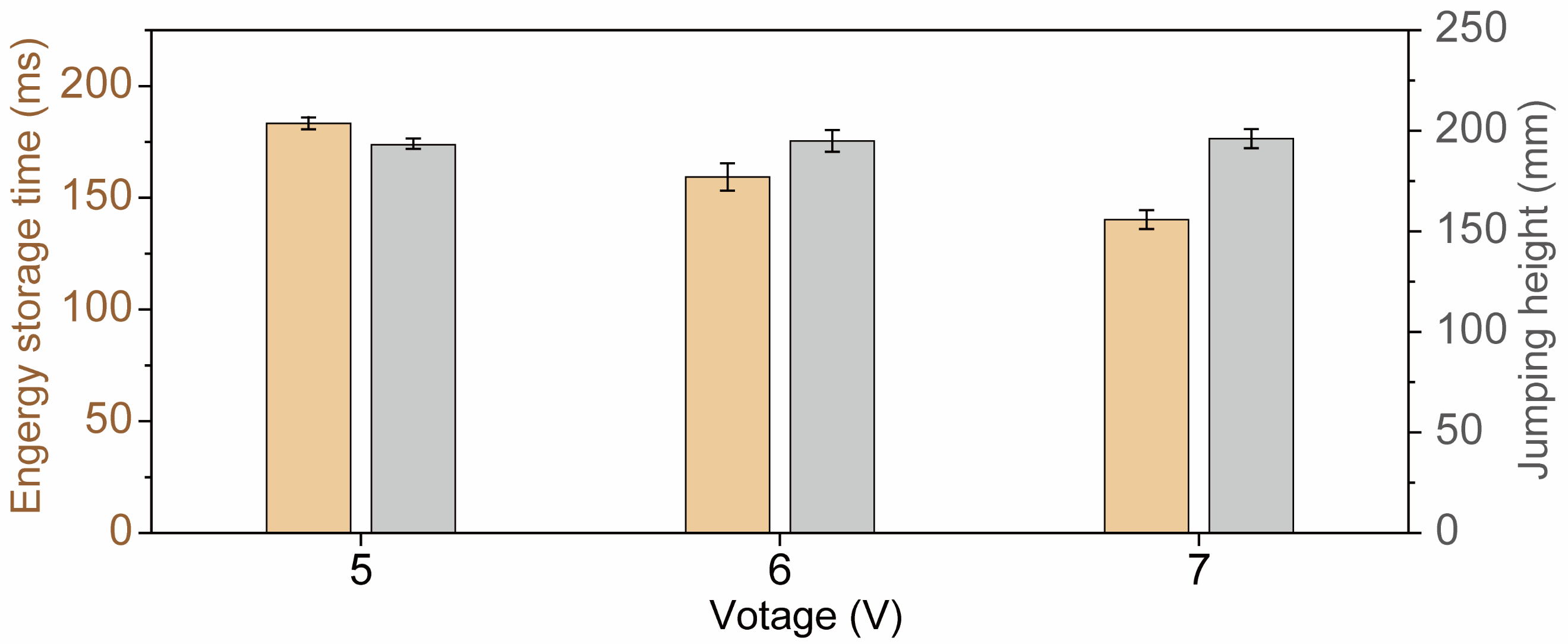


**Figure S14. Energy storage time and jumping height of the BATE jumper at varying air pump voltages.**


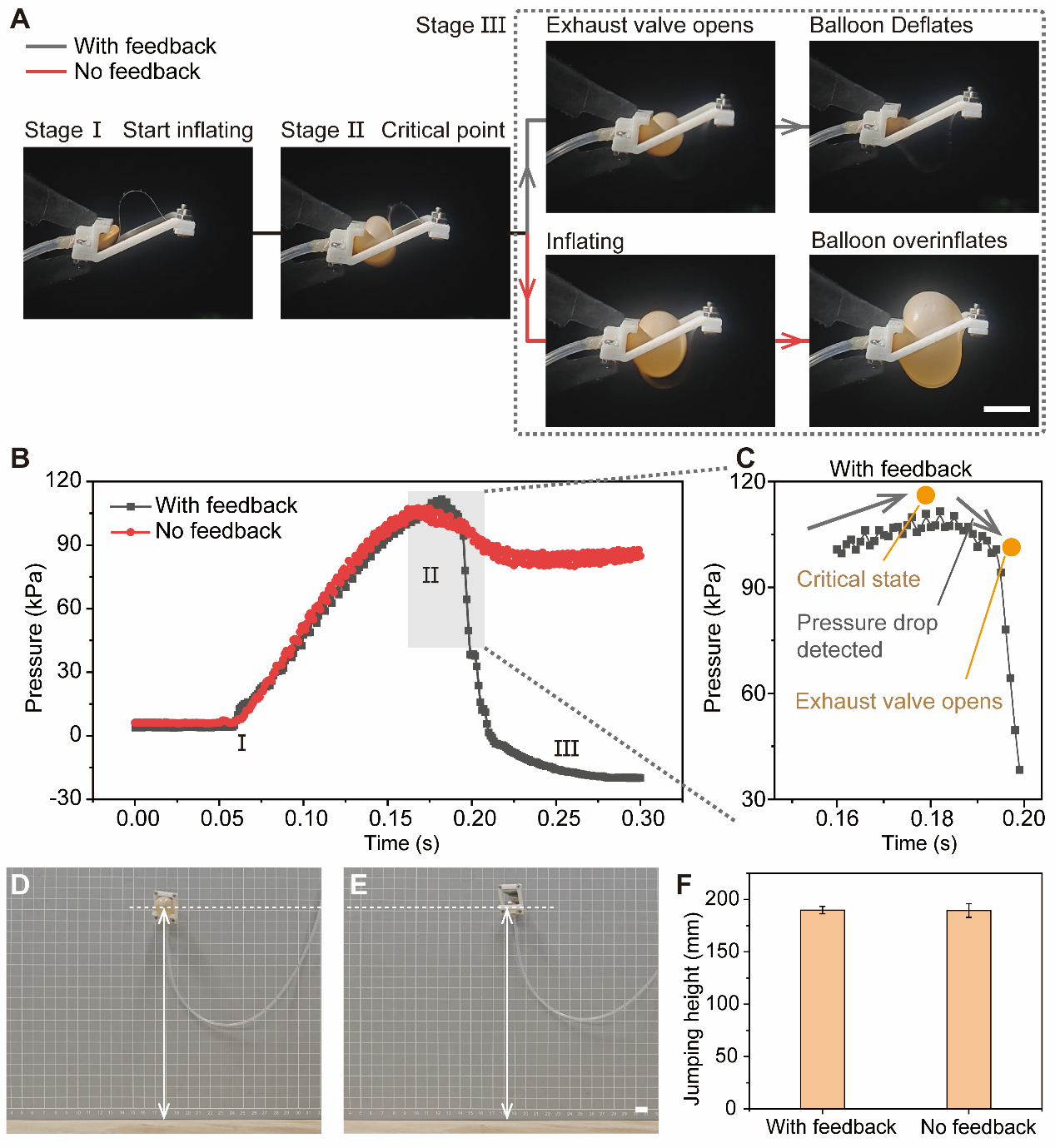


**Figure S15. A**) Video frames depicting the airbag actuation process of the bistable buckling beam with and without the feedback system. **B,C**) Internal pressure changes in the airbag during the actuation of the bistable beam with and without a feedback system. **D,E** and **F**) Performance comparison of the BATE jumper in terms of jumping height with and without feedback system.


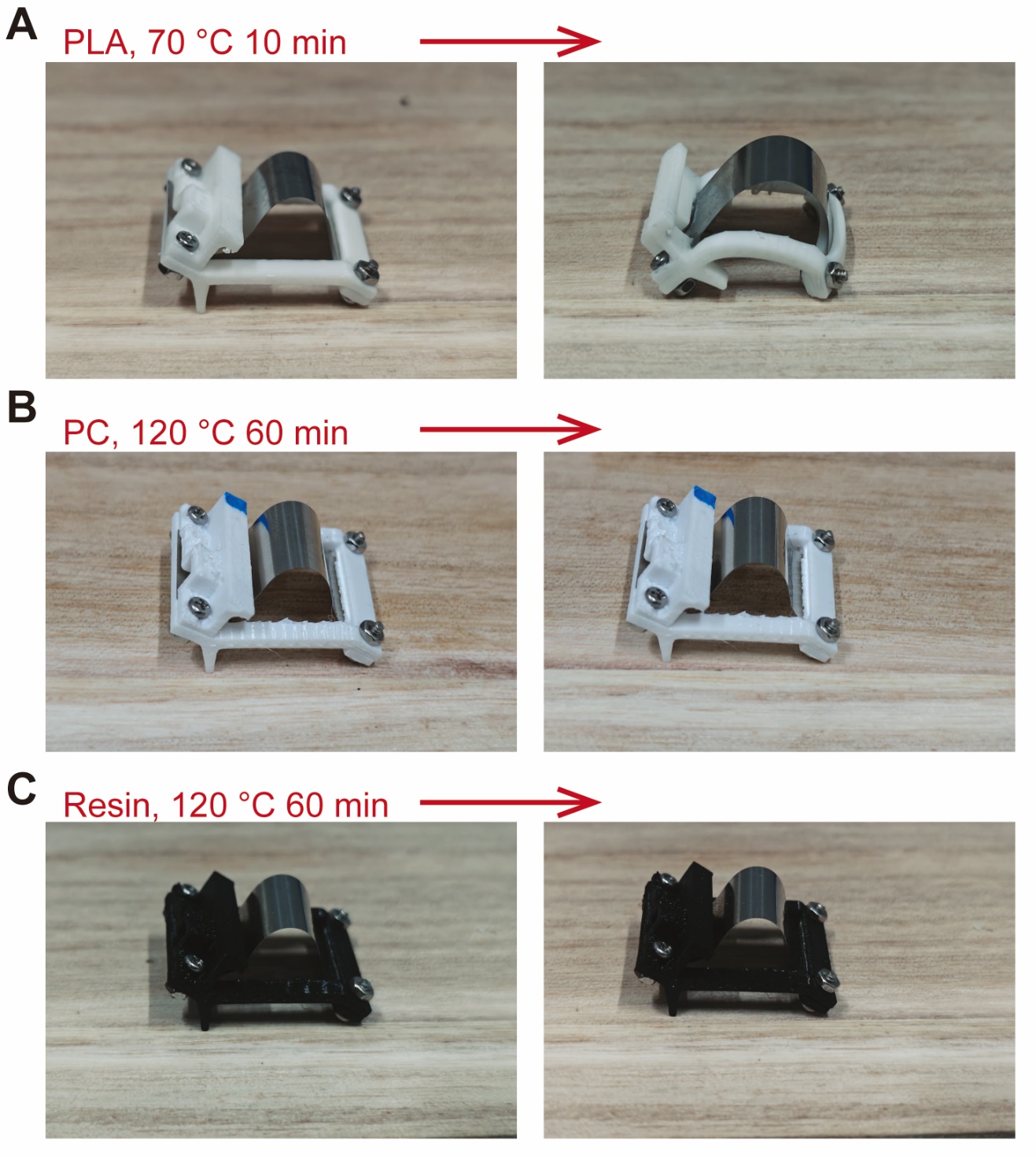


**Figure S16. A, B** and **C)** Deformation of three 3D printed body framework materials (i.e., PLA, PC and resin) under high-temperature environment ranging 60 °C to 120 °C.


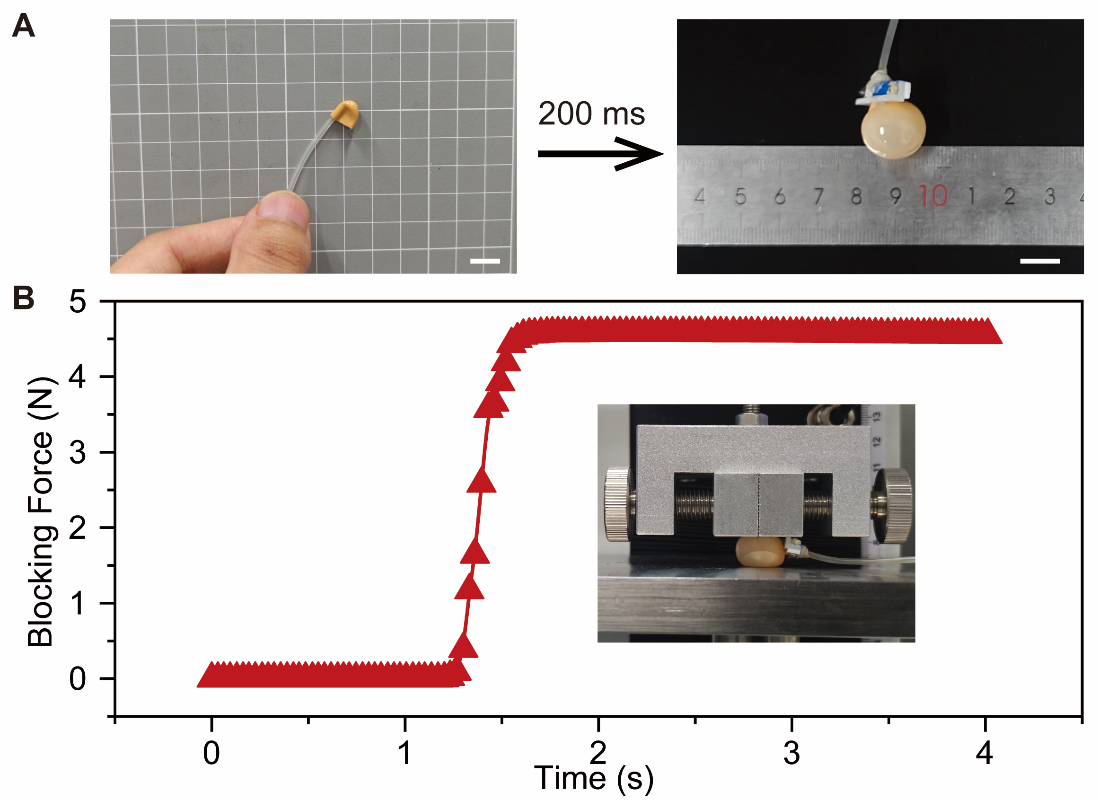


**Figure S17. A**) The original size of the airbag and its size after 200 ms of inflation. Scale bar, 10 mm. **B**) The blocking force of the airbag at a stroke of 10 mm.


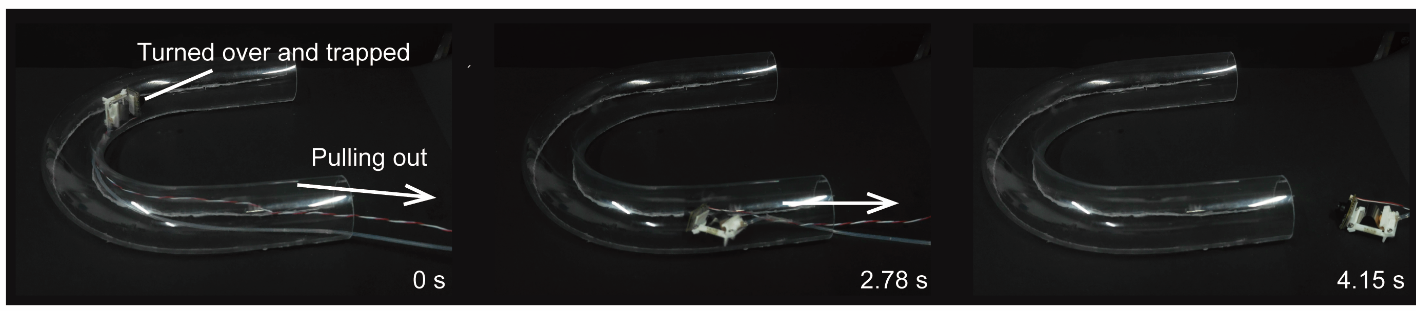


**Figure S18. Illustration of the BATE jumper being retrieved from a confined U-shaped tube by pulling the air tube.**

**Table S1.** Performance comparison of jumping robots in the literature.

| **S/N** | **Jumping robot (JR)** | **Date (year)** | **Refer.** | **Actuation methods** | **Body length (cm)** | **Weight (g)** | **JD (BL)** | **JH (BL)** | **JS**  **(m/s)** | **Continuous jumping capacity** |
| --- | --- | --- | --- | --- | --- | --- | --- | --- | --- | --- |
| 1 | TAUB | 2015 | (46) | Motor and spring | 13.5 | 23 | 10.14 | 24.8 | 9 | Yes |
| 2 | Miniature 7g JR | 2008 | (47) | Motor and spring | 5 | 7 | 15.8 | 27.6 | 4.2 | No |
| 3 | Single-Motor-Actuated JR | 2013 | (48) | Motor and spring | 6.5 | 23.5 | 13.8 | 13.4 | 4.3 | Yes |
| 4 | Work multiplication JR | 2022 | (9) | Motor and spring | 40 | 30.37 | N/A | 82.25 | 28 | Yes |
| 5 | Multi-locomotion millirobots | 2019 | (49) | SMA | 5.8 | 9.7 | 3.97 | 2.5 | 1.44 | Yes |
| 6 | Beetle-inspired JR | 2020 | (50) | SMA | 5 | N/A | N/A | 8 | N/A | No |
| 7 | Electrically-latched Jumper | 2019 | (51) | DEA | 5 | 2 | N/A | 1 | N/A | Yes |
| 8 | Hopping-Running Robot | 2019 | (52) | DEA | 8.5 (BH: 50 cm) | 6.5 | 0.29 | 0.25 | 0.5183 | Yes |
| 9 | Multi-modal Locomotion JR | 2020 | (53) | DEA | 6 | 0.9 | 1.34 | 1.16 | 0.223 | Yes |
| 10 | LSJR | 2021 | (5) | HASEL | 7 | 1.1 | 1.46 | 0.4 | 0.39 | Yes |
| 11 | 1 mm-Thick Soft Robot | 2020 | (55) | Pneumatic | 3.5 | 0.45 | 0.9 | 0.8 | 0.031 | Yes |
| 12 | Shell snapping JR | 2020 | (29) | Pneumatic | 6 | N/A | N/A | 4.71 | N/A | No |
| 13 | Electromagnetic robots | 2022 | (56) | Magnetic | 0.6 | 0.096 | N/A | 0.67 | N/A | Yes |
| 14 | Untethered, Roly-Poly JR | 2015 | (57) | Combustion | 18 | 2100 | 2.78 | 1.11 | 0.24 | Yes |
| 15 | 3D-printed, combustion JR | 2015 | (35) | Combustion | 30 | 964.6 | 0.5 | 2.5 | N/A | Yes |
| 16 | Combustion actuators | 2023 | (15) | Combustion | 2.9 | 1.6 | 5.3 | 20.34 | 0.17 | Yes |
| 17 | Robust Jumping Actuator | 2021 | (16) | Vapor | 1 | N/A | N/A | 2.6 | 0.71 | Yes |
| 17 | Polyacrylamide Hydrogel Actuators | 2023 | (18) | Moisture | 0.9 | 0.0034 | N/A | 5.9 | 0.13 | Yes |
| 18 | Dual-responsive jumping actuators | 2022 | (17) | Photothermal | 2 | N/A | 1.88 | 0.225 | 0.056 | Yes |
| 19 |  |  |  | Moisture | 1.5 | N/A | 2.5 | 0.88 | 0.17 | Yes |
| 20 | This work | * | | Pneumatic | 1.4 | 0.7^i^ | 6.1 | 13.6 | 1.8 | No |
|  |  |  |  |  |  | 0.9^ii^ | 21.4 | 10 | 0.8 ^a)^ | No |
|  |  |  |  |  | 1.9 | 1.2^iii^ | 4.2 | 1.3 | 0.13 ^b)^ | Yes |

(Notes: BATE jumper’s performance of height-, distance- and continuous-jump modes are marked with i-iii. a) Horizontal speed. b) Continuous jumping speed. JD jumping distance, BL body length, BH body height, JH jumping height and JS jumping speed.)

**Table S2.** Jumping performance comparison of insects in the literature.

| **S/N** | **Animals** | **Strategy** | **Body Length (mm)** | **Body Mass (mg)** | **Take-off Velocity (m/s) v** | **Jumping Distance (BL)** | **Jumping Height (BL)** | **Refer.** |
| --- | --- | --- | --- | --- | --- | --- | --- | --- |
| 1 | Schistocerca gregaria Locust | Catapult mechanism | 50 -80 | 70 | 3.2 | 19.70 | 3.48 | (58), (59) |
| 2 | Archaeopsyllus erinace Flea |  | 1.8 ± 0.19 | 0.7 ± 0.16 | 1.3 ± 0.21 | 148.91 | 18.89 | (60) |
| 3 | Philaenus Froghopper |  | 6.1 ± 0.08 | 12.3 ± 0.74 | 4.7 | 505.91 | 70.16 | (61) |
| 4 | Saltoblattella montistabularis Cockroach |  | 6.3 ± 10 | 31 | 1.5 ± 0.3 | 55.83 | 7.46 | (62) |
| 5 | Saldula saltatorial Shore bug |  | 3.5 ± 0.09 | 2.1 ± 0.09 | 1.3 ± 0.03 | 81.70 | 7.43 | (63) |
| 6 | Athous haemorrhoidalis Click beetle | Jumping without legs | 20.3 | 200 | 2.4 | 5.05 | 14.78 | (64) |
| 7 | Tomocerus longicornis Springtail |  | 2 | 2.1 | 1.4 | 15.00 | 10.00 | (65) |
| 8 | Trap jaw ants |  | ~10 | 13.5 | 7 | 20.00 | 7.20 | (25) |
| 9 | Sitticus pubescen Jump spider | Hydrostatic pressure | 6.1 ± 0.3 | 19.95 ± 4.71 | 0.99 ± 0.13 | 32.14 | 0.33 | (66) |

**Table S3.** Comparison of different bistable robots in terms of energy release and restorage time.

| S/N | **Bistable robot** | **Date (year)** | **Refer.** | **Power source** | **Body length (cm)** | **Energy release time （s）** | **Energy restorage time （s）** | **Total time (s)** |
| --- | --- | --- | --- | --- | --- | --- | --- | --- |
| 1 | Insect-scale jumping robots | 2023 | (30) | Heat | 2 | 0.0039 | 200 | 200.0039 |
| 2 | Soft and untethered robots | 2018 | (34) | Heat | 12 | 2.29 | 22.9 | 25.2 |
| 3 | Butterfly stroke–like soft swimmer | 2022 | (67) | Pneumatic | 15 | N/A | N/A | 1.403 |
| 4 | Spine-inspired soft robots | 2020 | (68) | Pneumatic | 7 ^a)^ | 0.98 | 0.98 | 1.96 |
| 5 | Multi-locomotion millirobots | 2019 | (49) | Heat | 5.8 | 4.14 | 6.28 | 10.42 |
| 6 | This work | * | | Pneumatic | 1.9 ^b)^ | 0.26 | 0.26 | 0.52 |
|  |  |  |  |  | 2.8 ^c)^ | 0.14 | 0.14 | 0.28 |

^a)^ Corresponding spring pretension length is 8 mm.

^b)^ We selected the BATE jumpers using the same configuration ([*h*, *v*] = [60, 50]%*S, S =* 15 mm) in Figure 1*.*

^c)^ BATE jumpers with the supply power of 7 V (refer to Supplementary Text 6, Supporting information).

**Table S4.** Comparison of different 3D printed body framework materials ^a)^.

| **Materials** | **Framework quality [g]** | **Working temperature [°C]** | **Unit price [RMB]** | **Preparation time [minutes]** | **Biodegradability** |
| --- | --- | --- | --- | --- | --- |
| PLA | 1.4 | < 60 | 0.11 (79 RMB/kg) | 20 | YES |
| PC | 1.4 | <120 | 0.31(219 RMB/kg) | 20 | NO |
| Resin | 1.3 | <120 | 1.12 (860 RMB/kg) | 135 (105 +30) ^b)^ | NO |

^a)^ Data from https://formlabs.com/materials/, https://bambulab.cn/zh-cn and JD.com.

^b)^ 105 minutes for printing and 30 minutes for UV light curing.

**Legends for Movies**

**Movie S1.** Force-displacement curves of symmetric bistable buckling beam triggered form center.

**Movie S2.** Force-displacement curves of antisymmetric bistable buckling beam triggered form boundaries.

**Movie S3.** BATE jumper in height, distance and continuous jump modes at an insect scale.

**Movie S4.** Close-up of BATE jumper in height-jump mode with ([*h, v*] = [60, 30]*%S*).

**Movie S5.** Close-up of BATE jumper in height-jump mode with ([*h, v*] = [60, 50]*%S*).

**Movie S6.** Body rotation during jump without counterweight and friction tape captured at 2000 fps.

**Movie S7.** High-speed camera close-up of snapping dynamic process of two antisymmetric equilibrium states.

**Movie S8.** Close-up of BATE jumper in distance-jump mode without counterweight and friction tape.

**Movie S9.** Close-up of BATE jumper in distance-jump mode with counterweight = 1.0 g and friction tape.

**Movie S10.** Jump cycle demonstration of the dual-airbag BATE jumper with continuous jump capability.

**Movie S11.** Continuous jumping demonstration on 35 mm steps.

**Movie S12.** Continuous jumping on flat wood substrate.

**Movie S13.** Steering capability demonstration of BATE jumper.

**Movie S14.** Camera-equipped BATE Jumper detects target objects by jumping over obstacles.

**Movie S15.** Monitoring system with real time feedback indication through different colored LEDs.

**References**

[1] R. M. Alexander, *Principles of Animal Locomotion*, Princeton University Press, **2003**.

[2] M. Burrows, *Nature* **2003**, *424*, 509.

[3] S. N. Patek, W. L. Korff, R. L. Caldwell, *Nature* **2004**, *428*, 819.

[4] D. Wei, W. Ge, *Int. J. Adv. Robot. Syst.* **2014**, *11*, 168.

[5] Z. Zhang, J. Zhao, H. Chen, D. Chen, *Appl. Bionics Biomech.* **2017**, *2017*, e4780160.

[6] M. H. Kaplan, H. S. Seifert, *J. Spacecr. Rockets* **1969**, *6*, 917.

[7] J. Burdick, P. Fiorini, *Int. J. Robot. Res.* **2003**, *22*, 653.

[8] W. Liu, F. Li, X. Fu, C. Stefanini, G. Bonsignori, U. Scarfogliero, P. Dario, in *Adv. Mechatron. MEMS Devices* (Ed.: D. Zhang), Springer, New York, NY, **2013**, pp. 207–221.

[9] E. W. Hawkes, C. Xiao, R.-A. Peloquin, C. Keeley, M. R. Begley, M. T. Pope, G. Niemeyer, *Nature* **2022**, *604*, 657.

[10] D. W. Haldane, M. M. Plecnik, J. K. Yim, R. S. Fearing, *Sci. Robot.* **2016**, *1*, eaag2048.

[11] A. Weiss, V. Zaitsev, N. Nabi, U. B. Hanan, *Eng. Res. Express* **2020**, *2*, 015017.

[12] M. A. Woodward, M. Sitti, *Int. J. Robot. Res.* **2014**, *33*, 1511.

[13] M. Kovač, M. Schlegel, J.-C. Zufferey, D. Floreano, *Auton. Robots* **2010**, *28*, 295.

[14] C. Tang, B. Du, S. Jiang, Q. Shao, X. Dong, X.-J. Liu, H. Zhao, *Sci. Robot.* **2022**, *7*, eabm8597.

[15] C. A. Aubin, R. H. Heisser, O. Peretz, J. Timko, J. Lo, E. F. Helbling, S. Sobhani, A. D. Gat, R. F. Shepherd, *Science* **2023**, *381*, 1212.

[16] K. Yu, X. Ji, T. Yuan, Y. Cheng, J. Li, X. Hu, Z. Liu, X. Zhou, L. Fang, *Adv. Mater.* **2021**, *33*, 2104558.

[17] J. Li, M. Wang, Z. Cui, S. Liu, D. Feng, G. Mei, R. Zhang, B. An, D. Qian, X. Zhou, Z. Liu, *J. Mater. Chem. A* **2022**, *10*, 25337.

[18] J. Li, G. Zhang, Z. Cui, L. Bao, Z. Xia, Z. Liu, X. Zhou, *Small* **2023**, *19*, 2303228.

[19] Y. Wu, J. K. Yim, J. Liang, Z. Shao, M. Qi, J. Zhong, Z. Luo, X. Yan, M. Zhang, X. Wang, R. S. Fearing, R. J. Full, L. Lin, *Sci. Robot.* **2019**, *4*, eaax1594.

[20] N. El-Atab, R. B. Mishra, F. Al-Modaf, L. Joharji, A. A. Alsharif, H. Alamoudi, M. Diaz, N. Qaiser, M. M. Hussain, *Adv. Intell. Syst.* **2020**, *2*, 2000128.

[21] J. Li, K. Yu, G. Wang, W. Gu, Z. Xia, X. Zhou, Z. Liu, *Adv. Funct. Mater.* **2023**, *33*, 2300156.

[22] J. M. McCracken, B. R. Donovan, T. J. White, *Adv. Mater.* **2020**, *32*, 1906564.

[23] O. Bolmin, J. J. Socha, M. Alleyne, A. C. Dunn, K. Fezzaa, A. A. Wissa, *Proc. Natl. Acad. Sci.* **2021**, *118*, e2014569118.

[24] X. Mo, W. Ge, M. Miraglia, F. Inglese, D. Zhao, C. Stefanini, D. Romano, *Appl. Sci.* **2020**, *10*, 8607.

[25] S. N. Patek, J. E. Baio, B. L. Fisher, A. V. Suarez, *Proc. Natl. Acad. Sci. U. S. A.* **2006**, *103*, 12787.

[26] N. Hu, B. Li, R. Bai, K. Xie, G. Chen, *Research* **2023**, *6*, 0116.

[27] Y. Chi, Y. Li, Y. Zhao, Y. Hong, Y. Tang, J. Yin, *Adv. Mater.* **2022**, 2110384.

[28] Y. Cao, M. Derakhshani, Y. Fang, G. Huang, C. Cao, *Adv. Funct. Mater.* **2021**, *31*, 2106231.

[29] B. Gorissen, D. Melancon, N. Vasios, M. Torbati, K. Bertoldi, *Sci. Robot.* **2020**, *5*, eabb1967.

[30] Y. Wang, Q. Wang, M. Liu, Y. Qin, L. Cheng, O. Bolmin, M. Alleyne, A. Wissa, R. Baughman, D. Vella, S. Tawfick, *Proc. Natl. Acad. Sci. U. S. A.* **2023**, *120*, e2210651120.

[31] J.-S. Koh, E. Yang, G.-P. Jung, S.-P. Jung, J. H. Son, S.-I. Lee, P. G. Jablonski, R. J. Wood, H.-Y. Kim, K.-J. Cho, *Science* **2015**, *349*, 517.

[32] H. Lee, C. Xia, N. X. Fang, *Soft Matter* **2010**, *6*, 4342.

[33] Y. Kim, J. van den Berg, A. J. Crosby, *Nat. Mater.* **2021**, *20*, 1695.

[34] T. Chen, O. R. Bilal, K. Shea, C. Daraio, *Proc. Natl. Acad. Sci.* **2018**, *115*, 5698.

[35] N. W. Bartlett, M. T. Tolley, J. T. B. Overvelde, J. C. Weaver, B. Mosadegh, K. Bertoldi, G. M. Whitesides, R. J. Wood, *Science* **2015**, *349*, 161.

[36] J. Jeon, J.-C. Choi, H. Lee, W. Cho, K. Lee, J. G. Kim, J.-W. Lee, K.-I. Joo, M. Cho, H.-R. Kim, J. J. Wie, *Mater. Today* **2021**, *49*, 97.

[37] Y. Yang, Y. Wang, *Adv. Sci.* **2024**, *11*, 2307088.

[38] B. Camescasse, A. Fernandes, J. Pouget, *Int. J. Solids Struct.* **2013**, *50*, 2881.

[39] W. Hartono, *ZAMM - J. Appl. Math. Mech. Z. Für Angew. Math. Mech.* **2001**, *81*, 605.

[40] J. Zhao, J. Jia, X. He, H. Wang, *J. Appl. Mech.* **2008**, *75*, DOI 10.1115/1.2870953.

[41] Z. P. Bazant, L. Cedolin, *Stability of Structures: Elastic, Inelastic, Fracture and Damage Theories*, World Scientific, **2010**.

[42] N. P. Linthorne, *Am. J. Phys.* **2001**, *69*, 1198.

[43] A. M. Wilson, J. C. Lowe, K. Roskilly, P. E. Hudson, K. A. Golabek, J. W. McNutt, *Nature* **2013**, *498*, 185.

[44] H.-W. Park, S. Park, S. Kim, in *2015 IEEE Int. Conf. Robot. Autom. ICRA*, **2015**, pp. 5163–5170.

[45] J. Zhang, G. Song, Y. Li, G. Qiao, A. Song, A. Wang, *Mechatronics* **2013**, *23*, 1123.

[46] V. Zaitsev, O. Gvirsman, U. B. Hanan, A. Weiss, A. Ayali, G. Kosa, *Bioinspir. Biomim.* **2015**, *10*, 066012.

[47] M. Kovac, M. Fuchs, A. Guignard, J.-C. Zufferey, D. Floreano, in *2008 IEEE International Conference on Robotics and Automation*, **2008**, pp. 373–378.

[48] J. Zhao, J. Xu, B. Gao, N. Xi, F. J. Cintrón, M. W. Mutka, L. Xiao, *IEEE Transactions on Robotics* **2013**, *29*, 602.

[49] Z. Zhakypov, K. Mori, K. Hosoda, J. Paik, *Nature* **2019**, *571*, 381.

[50] S.-M. Baek, S. Yim, S.-H. Chae, D.-Y. Lee, K.-J. Cho, *Science Robotics* **2020**, *5*, eaaz6262.

[51] M. Duduta, F. C. J. Berlinger, R. Nagpal, D. R. Clarke, R. J. Wood, F. Z. Temel, *Smart Mater. Struct.* **2019**, *28*, 09LT01.

[52] J. Zhao, J. Zhang, D. McCoul, Z. Hao, S. Wang, X. Wang, B. Huang, L. Sun, *Soft Robotics* **2019**, *6*, 713.

[53] M. Duduta, F. Berlinger, R. Nagpal, D. R. Clarke, R. J. Wood, F. Z. Temel, *IEEE Robotics and Automation Letters* **2020**, *5*, 3868.

[54] R. Chen, Z. Yuan, J. Guo, L. Bai, X. Zhu, F. Liu, H. Pu, L. Xin, Y. Peng, J. Luo, L. Wen, Y. Sun, *Nat Commun* **2021**, *12*, 7028.

[55] Z. Liu, J. Liu, H. Wang, X. Yu, K. Yang, W. Liu, S. Nie, W. Sun, Z. Xie, B. Chen, S. Liang, Y. Guan, L. Wen, *IEEE Robotics and Automation Letters* **2020**, *5*, 3291.

[56] G. Mao, D. Schiller, D. Danninger, B. Hailegnaw, F. Hartmann, T. Stockinger, M. Drack, N. Arnold, M. Kaltenbrunner, *Nat Commun* **2022**, *13*, 4456.

[57] M. Loepfe, C. M. Schumacher, U. B. Lustenberger, W. J. Stark, *Soft Robotics* **2015**, *2*, 33.

[58] Q.-V. Nguyen, H. C. Park, *J Bionic Eng* **2012**, *9*, 271.

[59] X. Mo, W. Ge, D. Romano, E. Donati, G. Benelli, P. Dario, C. Stefanini, *Entomologia Generalis* **2019**, 317.

[60] G. P. Sutton, M. Burrows, *Journal of Experimental Biology* **2011**, *214*, 836.

[61] M. Burrows, *Journal of Experimental Biology* **2006**, *209*, 4607.

[62] M. Picker, J. F. Colville, M. Burrows, *Biology Letters* **2011**, *8*, 390.

[63] M. Burrows, *Journal of Experimental Biology* **2009**, *212*, 106.

[64] M. E. G. Evans, *Journal of Zoology* **1973**, *169*, 181.

[65] J. Brackenbury, H. Hunt, *Journal of Zoology* **1993**, *229*, 217.

[66] T. Weihmann, M. Karner, R. J. Full, R. Blickhan, *J Comp Physiol A* **2010**, *196*, 421.

[67] Y. Chi, Y. Hong, Y. Zhao, Y. Li, J. Yin, *Science Advances* **2022**, *8*, eadd3788.

[68] Y. Tang, Y. Chi, J. Sun, T.-H. Huang, O. H. Maghsoudi, A. Spence, J. Zhao, H. Su, J. Yin, *Science Advances* **2020**, *6*, eaaz6912.
